# Release of HIV-1 sequestered in the vesicles of oral and genital mucosal epithelial cells by epithelial-lymphocyte interaction

**DOI:** 10.1371/journal.ppat.1006247

**Published:** 2017-02-27

**Authors:** Aizezi Yasen, Rossana Herrera, Kristina Rosbe, Kathy Lien, Sharof M. Tugizov

**Affiliations:** 1 Department of Medicine, University of California–San Francisco, San Francisco, California, United States of America; 2 Department of Otolaryngology, University of California–San Francisco, San Francisco, California, United States of America; University of Illinois at Chicago College of Medicine, UNITED STATES

## Abstract

Oropharyngeal mucosal epithelia of fetuses/neonates/infants and the genital epithelia of adults play a critical role in HIV-1 mother-to-child transmission and sexual transmission of virus, respectively. To study the mechanisms of HIV-1 transmission through mucosal epithelium, we established polarized tonsil, cervical and foreskin epithelial cells. Analysis of HIV-1 transmission through epithelial cells showed that approximately 0.05% of initially inoculated virions transmigrated via epithelium. More than 90% of internalized virions were sequestered in the endosomes of epithelial cells, including multivesicular bodies (MVBs) and vacuoles. Intraepithelial HIV-1 remained infectious for 9 days without viral release. Release of sequestered intraepithelial HIV-1 was induced by the calcium ionophore ionomycin and by cytochalasin D, which increase intracellular calcium and disrupt the cortical actin of epithelial cells, respectively. Cocultivation of epithelial cells containing HIV-1 with activated peripheral blood mononuclear cells and CD4+ T lymphocytes led to the disruption of epithelial cortical actin and spread of virus from epithelial cells to lymphocytes. Treatment of epithelial cells with proinflammatory cytokines tumor necrosis factor-alpha and interferon gamma also induced reorganization of cortical actin and release of virus. Inhibition of MVB formation by small interfering RNA (siRNA)-mediated silencing of its critical protein hepatocyte growth factor-regulated tyrosine kinase substrate (Hrs) expression reduced viral sequestration in epithelial cells and its transmission from epithelial cells to lymphocytes by ~60–70%. Furthermore, inhibition of vacuole formation of epithelial cells by siRNA-inactivated rabankyrin-5 expression also significantly reduced HIV-1 sequestration in epithelial cells and spread of virus from epithelial cells to lymphocytes. Interaction of the intercellular adhesion molecule-1 of epithelial cells with the function-associated antigen-1 of lymphocytes was important for inducing the release of sequestered HIV-1 from epithelial cells and facilitating cell-to-cell spread of virus from epithelial cells to lymphocytes. This mechanism may serve as a pathway of HIV-1 mucosal transmission.

## Author summary

Although the majority of HIV-1 transmissions occur through the mucosal epithelium, the mechanism of initial events of viral spread from epithelia to HIV-1-susceptible CD4+ lymphocytes, macrophages, and Langerhans/dendritic cells is not well understood. In this study we observed that >90% of virions internalized in tonsil, cervical and foreskin epithelial cells did not cross the epithelium but rather were retained in the endosomal compartments for several days. These sequestered virions in the epithelium were infectious and were released by the interaction of activated PBMC and CD4+ T lymphocytes with epithelial cells containing the virus. The specific interaction of lymphocyte receptor function-associated antigen-1 (LFA-1) with the epithelial cell receptor intercellular adhesion molecule-1 (ICAM-1) was critical for the induction of HIV-1 release and the cell-to-cell spread of HIV-1 from epithelial cells to lymphocytes. This finding may explain the initial step of HIV-1 transmission from mucosal epithelia to virus-susceptible lymphocytes, macropages and Langerhans/dendritic cells, which migrate into oral and genital epithelia and capture virus from epithelial cells.

## Introduction

Transmission of human immunodeficiency virus-1 (HIV-1) through mucosal epithelium plays a critical role in the initiation of systemic HIV-1 infection and the development of acquired immune deficiency syndrome (AIDS). The fetus/infant oropharyngeal mucosal epithelia serve as a portal of entry for HIV-1 mother-to-child transmission (MTCT) [1, 2]. Adult cervical and foreskin epithelia serve as an entry site for sexual transmission of HIV-1 [3–6]. One of the possible pathways of HIV-1 transmission through mucosal epithelium could be viral transcytosis, i.e., transcellular transport of virions by vesicular/endosomal machinary of epithelial cells. Transepithelial transcytosis has been shown in epithelial cells of oral, intestinal, vaginal and endometrial origin [1, 7–12]. However, cell-free HIV-1 transcytosis via mucosal epithelial cells is not highly efficient, i.e., 0.01–0.05% of virions from the initial inoculum may translocate across epithelial cells [1, 8, 9]. The fate of internalized but untranscytosed virus has been poorly investigated. Furthermore, very little is known about the specific role of the endosomal/vesicular transport machinery of mucosal epithelium in the regulation of HIV-1 transepithelial transport.

Mucosal epithelial cells have polarized the organization and highly specialized vesicular compartments for endocytosis, macropinocytosis, transcellular transport, storage and recycling of proteins, antigens, and viruses [13–18]. Initial viral internalization into epithelial cells may follow intracellular trafficking of virions via early and late endosomes and macropinosomes [19–21]. Early endosomes, also called sorting compartments, regulate delivery of internalized virus to various destinations, including the opposite side of polarized membrane of epithelial cells, by transcytosis [18, 22]. Late endosomes consist of multivesicular bodies (MVB) and lysosomes [23, 24]. Macropinosomes are generated by macropinocytosis, which is an actin-dependent process induced by membrane ruffling [17, 19]. Some subapical endosomes and macropinosomes may fuse with each other and form large vacuoles, which may exist independently from the early and late endosomes [21, 25–30].

HIV-1 has been detected in the early and late endosomes and MVB of epithelial cells [1, 12, 31–34]. MVBs are spherical and nonacidic vesicular compartments that may serve as intracellular storage for proteins and viruses [35–39]. MVBs can fuse with plasma membranes, releasing intravesicular content (cargo) into the extracellular environment, i.e., exocytosis [24, 27, 40–45]. The elevation of intracellular calcium is critical for endosomal trafficking and the fusion of endosomes with plasma membranes [27, 46]. Actin cortex consisting of the actin cytoskeleton network connected to the plasma membrane [47] may serve as a barrier to fusion of endosomal membranes with the cell membrane, blocking the release of endosomal contents [45, 48–50].

Oral and genital mucosal epithelia are stratified and contain intraepithelial CD4+ lymphocytes, Langerhan/dendritic cells and macrophages [51–54], which directly interact with epithelial cells through adhesion molecules. Intercellular adhesion molecule-1 (ICAM-1) is a cell surface glycoprotein expressed in endothelial and epithelial cells, including oral, bronchial, skin, kidney, and intestinal epithelia [55–58]. Lymphocyte receptor function-associated antigen-1 (LFA-1) is a β-2 integrin consisting of an α chain (CD11a) and a β chain (CD18) and expressed in all white blood cells, including CD4+ lymphocytes, Langerhan/dendritic cells, and macrophages [59–62]. LFA-1 plays a critical role in the migration of lymphocytes, Langerhan/dendritic cells and macrophages across endothelial and epithelial cells [60, 62]. The interaction of LFA of lymphocytes, Langerhan/dendritic cells and macrophages with epithelial ICAM-1 is critical for adhesion of lymphocytes to epithelial cells [58, 63–68].

In the work presented here, we show that in polarized tonsil, cervical and foreskin epithelial cells, multiple HIV-1 virions are sequestered within the MVB and vacuoles for several days without release. The interaction of activated peripheral blood mononuclear cells (PBMC) and CD4+ T lymphocytes with epithelial cells containing sequestered HIV-1 through LFA-1 of lymphocytes and ICAM-1 of epithelial cells induces disruption of the cortical actin cytoskeleton and the spread of intraepithelial virions to lymphocytes.

## Results

### HIV-1 intracellular retention in polarized mucosal epithelial cells

To compare transcytosed HIV-1 with untranscytosed intracellular virions, we added dual-tropic HIV-1_SF33_ to the apical (AP) surface of polarized tonsil, cervical and foreskin epithelial cells. After 1, 3, 6 and 9 days, culture medium from the lower chambers of Transwell inserts and trypsinized cells were examined by HIV-1 ELISA p24 assay for transcytosed and intracellular virus, respectively.

**Fig 1:**
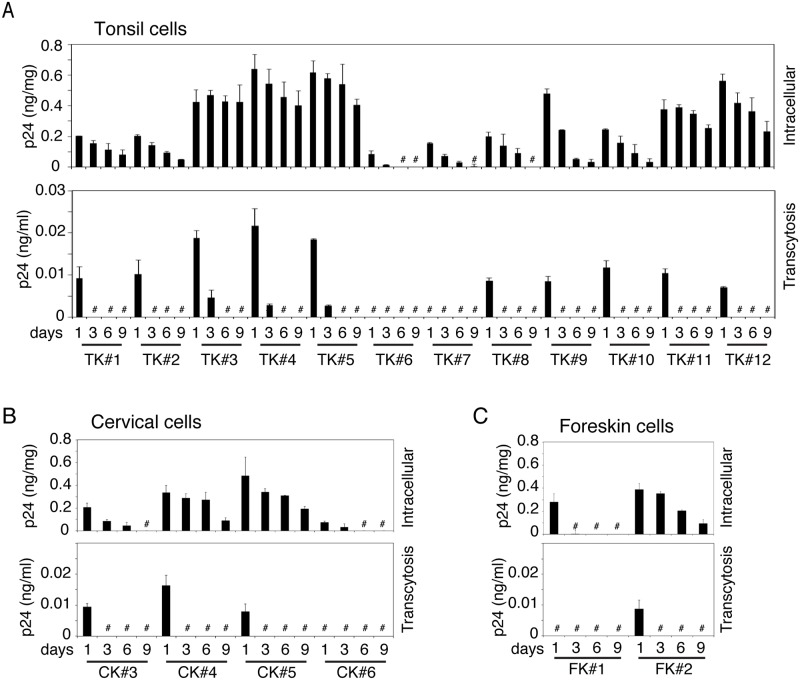
Analysis of transcytosed and intracellular virions in polarized tonsil, cervical and foreskin epithelial cells. HIV-1_SF33_ (20 ng/ml; p24) was added to the AP surface of polarized tonsil (A), cervical (B), and foreskin (C) epithelial cells, and 4 h later uninternalized virions were removed with trypsin. At days 1, 3, 6 and 9 after inoculation of virus, the culture medium from lower chambers and trypsinized cells were collected for detection of transcytosed virions (lower panels) and intracellular virus (upper panels). Transcytosed and intracellular virions were examined by ELISA p24. # Not detected. Data are shown as mean ± SEM (bars) of triplicate values.

Transcytosis of HIV-1 was detected in 10 of 12 (80%) tonsil, 3 of 4 (75%) cervical and 1 of 2 (50%) foreskin epithelial cells at day 1 (Fig 1A–1C, lower panels). Three of 12 (25%) tonsil cells also showed viral transcytosis at day 3. Virus release was not detected in these cells at any other time. Approximately 3% of intracellular virus was transcytosed from BL membranes of polarized tonsil, cervical and foreskin epithelial cells, indicating that ~97% of virions were retained in the cells.

Analysis of untranscytosed intracellular HIV-1 showed that in tonsil epithelial cells, 11 of 12 primary cultures (91%) contained intracellular virus (Fig 1A, upper panel). Of the 11 cell cultures, 9 contained intracellular virions for up to 9 days, and 2 contained intracellular virions for 6 days. In most of the cell lines, the concentration of intracellular HIV-1 was highest at day 1 and gradually decreased. Two of 4 cervical keratinocyte cell cultures maintained intracellular HIV-1 for up to 9 days, and one contained virus for 6 days (Fig 1B, upper panel). One of 2 foreskin keratinocytes contained intracellular HIV-1 for up to 9 days (Fig 1C, upper panel).

### Intraepithelial HIV-1 is infectious

**Fig 2:**
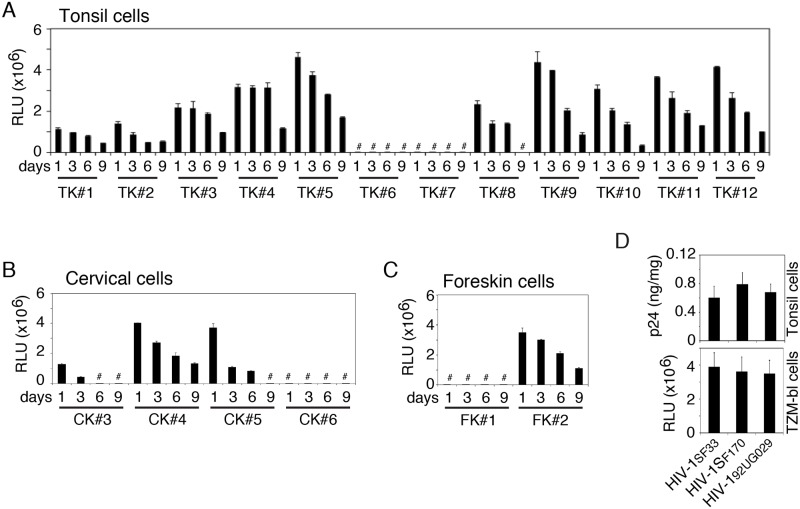
Intracellular HIV-1 preserves infectious activity. HIV-1_SF33_ was added to the AP surface of polarized tonsil (A), cervical (B) and foreskin (C) epithelial cells. After 1, 3, 6 or 9 days, cells were trypsinized and infectivity of intracellular virions was examined in TZM-bl cells. Data are shown as mean ± SEM of triplicate values. # Not detected. (D) Dual-tropic HIV-1_SF33_, R5-tropic HIV-1_SF170_ and X4-tropic HIV-1_92UG029_ viruses were added to the AP surface of polarized tonsil epithelial cells. (Upper panel) At day 6 one set of cells were trypsinized and examined for intracellular virions by ELISA p24. (Lower panel) The second set of cells were examined for infectivity of intracellular virions in TZM-bl cells. Data are expressed as mean ± SEM of three independent experiments (in triplicate) (n = 3). RLU: relative light units.

We examined HIV-1_SF33_ infectivity of intracellular virions in TZM-bl cells. Nine of 12 tonsil epithelial cell cultures contained infectious virions until 9 days after inoculation (Fig 2A). One culture contained infectious virions for up to 6 days. Intracellular virions from 2 of 4 cervical epithelial cell cultures (Fig 2B) and 1 of 2 foreskin epithelial cell cultures (Fig 2C) were also infectious.

To compare the intraepithelial retention of dual X4/R5-, X4- and R5-tropic strains of HIV-1 and their infectivity, we exposed polarized tonsil epithelial cells from 3 independent donors to dual-tropic HIV-1_SF33_, R5-tropic HIV-1_SF170_ and X4-tropic HIV-1_92UG029_ viruses. After 6 days, cells were tested for intracellular virions, which showed that intracellular retention of all 3 viruses was comparable in three epithelial cell cultures (Fig 2D, upper panel). Analysis of infectivity of these viruses in TZM-bl cells showed that intraepithelial virions of X4/R5-, X4- and R5-tropic strains of HIV-1 in all 3 cells were infectious (Fig 2D, lower panel).

### HIV-1 localization in early and late endosomes and vacuoles

We have seen that a majority of internalized virions in polarized tonsil, cervical and foreskin epithelial cells were retained in the cells. The lack of viral release at 6–9 days suggested that intracellular virions might be sequestered in the vesicular/endosomal compartments. To determine HIV-1 localization in the endosomes, HIV-1_SF33_ was added to the AP surface of polarized tonsil epithelial cells, which were incubated for 30 min at 37°C and fixed. In parallel experiments, cells containing virus were cultured for 6 days. Fixed cells were costained for HIV-1 p24 and for early and late endosome markers. For detection of early endosomes, cells were immunostained for early endosome antigen 1 (EEA1) [69]. For detection of late endosomes, we used antibodies against lysobisphosphatidic acid (LBPA) and lysosome-associated membrane protein 1 (LAMP1), which are markers for MVB and lysosomes, respectively [70–73]. The vacuoles were stained with their marker rabankyrin-5 [21].

**Fig 3:**
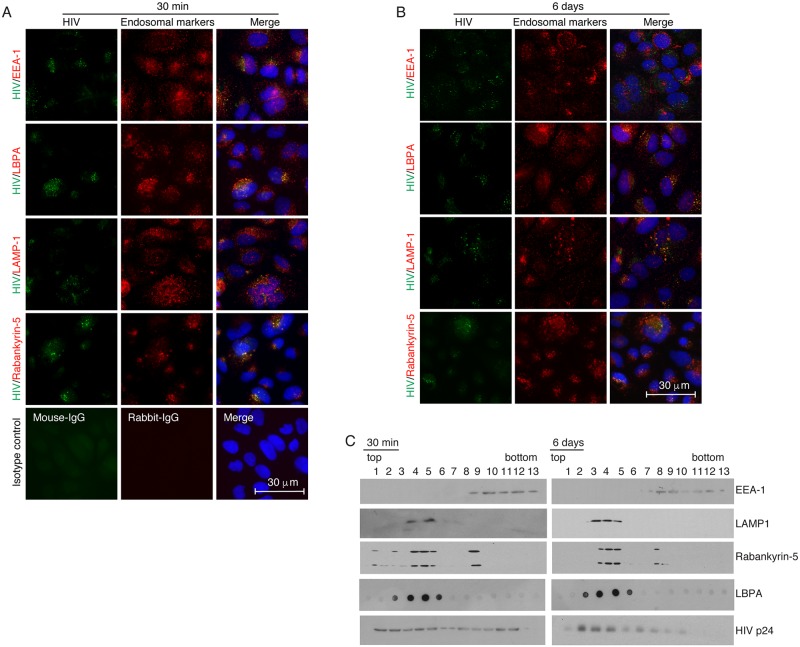
Penetration of HIV-1 in early and late endosomes, and vacuoles. (A) HIV-1_SF33_ was added to the AP surface of polarized tonsil epithelial cells, and one set of cells was fixed after 30 min. (B) Another set of cells were cultured for 6 days after removing uninternalized virions. Cells were coimmunostained for HIV-1 p24 (green) and EEA1, LBPA, LAMP1 and rabankyrin-5 (all red), which are markers for early endosomes, MVBs, lysosomes, and vacuoles, respectively. (A, bottom panels). Cells were stained with mouse and rabbit isotype control antibodies. Cells were analyzed by Nikon Eclipse E400 fluorescence microscope. Yellow indicates colocalization of HIV-1 p24 with endosome markers. Nuclei are counterstained with DAPI (blue). Similar data were obtained in three independent experiments. (C) HIV-1_SF33_ (200 ng/ml p24) was added to the AP surface of polarized tonsil cells grown in Transwell inserts with a 24-mm diameter. One set of cells were cultured for 30 min, and another set of cells were cultured for 6 days. Cells were dissociated with trypsin, and homogenized and vesicular fractions were separated in sucrose gradients. Each fraction was examined for EEA1, LAMP1, rabankyrin, LBPA, and HIV-1 p24 by Western blotting. Similar data were reproduced in two independent experiments. Immunoblots were performed at least twice and representative results are shown.

Immunofluorescence microscopy showed that HIV-1_SF33_ was colocalized with EEA1 of tonsil cells after 30 min of viral inoculation (Fig 3A); however, we did not observe viral colocalization with EEA1 after 6 days (Fig 3B). HIV-1 colocalization with late endosome markers LBPA and LAMP1, and vacuole marker rabankyrin-5 was detected at 30 min and at 6 days. Virions were detected in approximately 30% of cells, and intravesicular HIV-1 was detected as a cluster of virions in the early and late endosomes and vacuoles.

To determine HIV-1 sequestration in purifed early and late endosomal compartments, we added HIV-1 to the AP surface of tonsil epithelial cells. After 30 min or 6 days, 13 vesicular fractions were separated in a sucrose gradient. Early and late endosomes containing HIV-1 were examined by Western blot assay using antibodies against EEA1, LAMP1, LBPA and vacuoles. EEA1-positive early endosomes were detected mostly between 9 and 13 fractions, and LAMP1- and LBPA-positive late endosomes were detected between 3 and 7 fractions (Fig 3C). A small portion of rabankyrin-5 was identified in the early endosomes but was predominantly present in the late endosomes. The MVB marker LBPA was detected exclusively in late endosomes. Thirty minutes after inoculation of HIV-1, viral p24 was detected in both early and late endosomes (Fig 3C, left panels). After 6 days, most HIV-1 was detected in late endosomes (Fig 3C, right panels), indicating that viral sequestration occurs predominantly in late endosomes, including LBPA- and rabankyrin-5-positive MVB and vacuoles, respectively.

### Release of HIV-1 from epithelial cells by treatment with ionomycin and cytochalasin D

**Fig 4:**
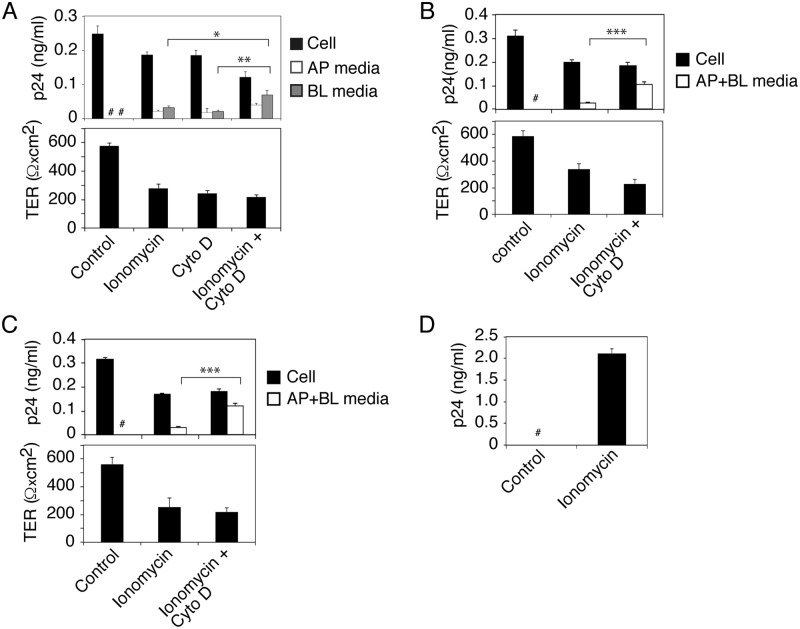
Induction of HIV-1 release from sequestration in epithelial cells. (A) Polarized tonsil epithelial cells containing HIV-1_SF33_ for 6 days were treated with ionomycin (10 μM), cytochalasin D (12 μg/ml), or a combination of ionomycin and cytochalasin D for 30 min. Intracellular virus and virus released from AP and BL medium were examined by ELISA p24 (upper panel) and TER was measured in control and treated cells (lower panel). Mean values ± SEM of three independent experiments (in triplicate or quadruplicate) are shown (n = 3). *P < 0.01 and **P < 0.002, BL virus release of ionomycin+cytochalasin D-treated cells compared with BL virus release of cells treated independently with ionomycin or cytochalasin D. (B, C) Polarized tonsil epithelial cells containing sequestered R5-tropic HIV-1_SF170_ (B) and X4-tropic HIV-1_92UG029_ (C) at day 6 were treated with ionomycin or a combination of ionomycin and cytochalasin D for 30 min. TER of polarized cells was measured in control and treated cells. Combined AP and BL medium and trypsinized cells were examined for released and intracellular virus, respectively. Mean values ± SEM of three independent experiments (in triplicate) are shown (n = 3). ***P < 0.0001, virus release from cells treated with ionomycin+cytochalasin D compared with ionomycin alone. (D) Polarized tonsil epithelial cells containing sequestered HIV-1_SF33_ at day 6 were treated with ionomycin for 30 min or untreated. Activated CD4+ T lymphocytes were infected with culture medium collected from untreated and ionomycin-treated cells. After 10 days, CD4+ T lymphocytes were examined for HIV-1 infection by using ELISA p24. Results were reproduced in three independent experiments and are shown as mean ± SEM (n = 3). # Not detected.

The lack of release of intravesicular virions suggested that fusion of endosomes containing HIV-1 with plasma membranes may require specific intracellular and/or extracellular signals for exocytosis of virus. For example, exocytosis required a rapid increase in intracellular calcium [27, 46] and disruption/reorganization of the cortical actin cytoskeleton [46, 74, 75]. To examine the roles of intracellular calcium elevation and cortical actin disruption in HIV-1 release, we treated polarized tonsil epithelial cells containing HIV-1_SF33_ with ionomycin (10 μM) or cytochalasin D (12 μg/ml), which induce intracellular calcium [27, 46] and disrupt cortical actin [76], respectively. One set of cells were treated with a combination of ionomycin and cytochalasin D. After 30 min, culture medium from AP and BL membranes was examined for released virus; results showed that both ionomycin and cytochalasin D induced HIV-1 release from AP and BL membranes (Fig 4A, upper panel). A combination of ionomycin and cytochalasin D significantly increased BL viral release by ~2.5-3-fold compared to BL release through independent treatment of cells with either one alone. Release of virus was correlated with reduction of intracellular virus. Analysis of transepithelial resistance (TER) showed that ionomycin and cytochalasin D and their combination reduced TER by approximately 50–60%, indicating depolarization of tonsil epithelial cells (Fig 4A, lower panel).

In parallel experiments we examined TER, paracellular permeability and cell viability of ionomycin- and/or cytochalasin D-treated and untreated (control) polarized tonsil epithelial cells (S1 Fig). To induce the paracellular permeability of polarized cells, we treated one set of cells with 10 mM EDTA, which disrupts epithelial junctions by opening their paracellular space [77, 78]. Data showed that ionomycin and/or cytochalasin D treatment did reduce TER (S1 Fig, upper panel) but did not induce paracellular permeability of polarized cells (S1 Fig, middle panel), indicating that the reduction of TER by these drugs was not sufficient to induce paracellular permeability (S1 Fig, middle panel). Paracellular leakage was seen only in EDTA-treated cells, which was correlated with a substantial reduction in TER. Since paracellular leakage was not detected by ionomycin and/or cytochalasin D treatment, the BL release of HIV-1 was not due to paracellular passage of virus. Measurement of cell viability showed that ionomycin and cytochalasin B alone and in combination did not have a toxic effect on the cells (S1 Fig, bottom panel).

To examine the inducible release of sequestered X4 and R5 strains of HIV-1, we treated polarized tonsil epithelial cells containing intracellular R5-tropic HIV-1_SF170_ and X4-tropic HIV-1_92UG029_ with ionomycin alone and in combination with cytochalasin D for 30 min (Fig 4B and 4C). Analysis of combined AP and BL medium showed that the release of both R5-tropic HIV-1_SF170_ and X4-tropic HIV-1_92UG029_ was inducible by ionomycin and combined ionomycin/cytochalasin B treatment, in contrast to control cells (Fig 4B and 4C, upper panels). Virus release was significantly higher with ionomycin/cytochalasin D than with ionomycin alone. Virus release was correlated with the reduction of intracellular virions (Fig 4B and 4C, upper panels) and TER (Fig 4B and 4C, lower panels).

Incubation of activated CD4 T lympocytes with culture medium of ionomycin-treated tonsil epithelial cells containing virus showed that the released virions were infectious (Fig 4D). Culture medium from untreated tonsil cells did not infect CD4+ T lymphocytes.

### Interaction of activated PBMC and CD4+ T lymphocytes with epithelial cells containing HIV-1 reduces TER of epithelial cells and induces release of sequestered HIV-1

**Fig 5:**
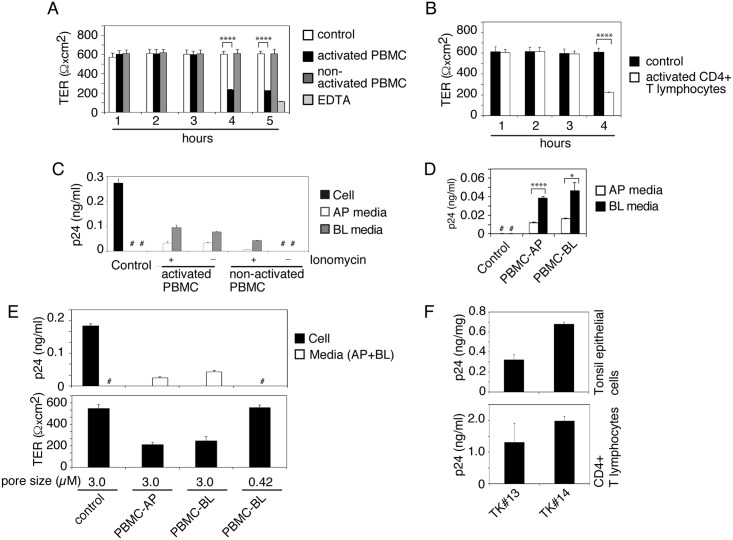
Cocultivation of activated PBMC and CD4+ T lymphocytes with epithelial cells containing HIV-1 induces virus release. (A) Activated and nonactivated PBMC were added to the AP surface of polarized tonsil epithelial cells (2:1), and after 1, 2, 3, 4 and 5 h the TER was examined. As a control, one set of polarized tonsil cells were not cocultivated. Another set of cells were treated with 10 mM EDTA for 30 min. (B) Activated CD4+ T lymphocytes were added to the AP surface of tonsil epithelial cells, and after 4 h the TER was examined. (A, B) Mean values ± SEM of three independent experiments (in triplicate) are shown (n = 3). ****P < 0.00001, TER of polarized cells cocultivated with activated PBMC or CD4+ T lymphocytes compared with TER of control cells. (C) Activated and nonactivated PBMC with or without ionomycin were added to the BL surface of tonsil cells containing intracellular HIV-1_SF33_ at day 6. After 4 h of cocultivation AP and BL medium was collected separately, centrifuged, and supernatants were examined for released virus using ELISA p24. Polarized epithelial cells and culture medium without cocultivation with PBMC were used as a control. (D) Activated PBMC were added to the AP or BL surface of tonsil cells containing HIV-1. Four hours later, AP and BL medium was examined for HIV-1 p24. (E) Polarized tonsil epithelial cells were grown in Transwell inserts with 3-μm or 0.42-μm pore size and exposed to HIV-1_SF33_ from AP membranes. Epithelial cells with sequestered virus at day 6 were cocultured with activated PBMC from the AP surface of polarized cells grown in inserts with 3.0-μm pore size and from BL membranes in inserts with 3-μm or 0.42-μm pore size. After 4 h, the TER of polarized cells was measured, and the culture medium from AP and BL membranes were combined for ELISA p24. (F). Matching tonsil keratinocytes and CD4+ T lymphocytes were isolated from the same tonsil of 2 independent donors. HIV-1_SF33_ was added to the AP surface of polarized tonsil epithelial cells, and at day 6 intracellular virus was examined by ELISA p24 (F, upper panel). One set of cells containing virus were used for cocultivation of autologous CD4+T lymphocytes. After 4 h, lymphocytes were collected, grown for 12 days and examined by ELISA p24 (F, lower panel). Data in panels C-F represent one of two or three independent experiments and are shown as mean ± SEM of triplicate values. (D) *P < 0.01, ****P < 0.00007, BL release of virus compared with AP release.

To study the spread of sequestered HIV-1 from epithelial cells into target cells, we cocultured epithelial cells containing HIV-1 with PBMC. To determine whether cocultivation of epithelial cells with lymphocytes reduces TER of epithelial cells, we added activated or nonactivated PBMC to the AP surface of polarized tonsil epithelial cells. One set of cells were treated with EDTA for 30 min. The TER of polarized cells was examined after 1, 2, 3, 4 and 5 h of PBMC cocultivation. These data showed that PBMC cocultivation with epithelial cells did not reduce the TER of polarized cells at 1, 2 and 3 h after cocultivation (Fig 5A). However, ~60% of TER reduction was detected after 4 and 5 h of cocultivation. A substantial reduction in TER (~90%) was detected by EDTA treatment. Reduction of epithelial TER was detected only with cocultivation of activated PBMC, in contrast to nonactivated PBMC, which did not change the TER.

Next, we measured the paracellular permeability of polarized cells cocultivated with PBMC (S2A Fig, upper panel), which showed a lack of paracellular leakage after any time point of cocultivation of epithelial cells with activated or nonactivated PBMC. EDTA drastically reduced TER (by ~90%) and induced paracellular leakage. Analysis of cell viability showed that cocultivation of epithelial cells with activated and nonactivated PBMC did not cause cell death (S2A Fig, lower panel).

We also examined the TER of polarized tonsil epithelial cells cocultivated with activated CD4+ T lymphocytes, and TER was reduced by ~60% after 4 h of cocultivation (Fig 5B). Reduction of epithelial TER by activated PBMC and CD4+ T lymphocytes was dependent on the lymphocyte–epithelial ratio (S2B Fig). Interaction of lymphocytes with epithelial cells at 1:1 and 1:2 ratios reduced the TER by ~50%, in contrast to the 1:10 ratio, which reduced TER by ~20%. TER reduction was not detectable at a lymphocyte–epithelial ratio of 1:20.

Lymphocytes migrate from lamina propria into mucosal epithelium [53] and may interact with the BL membranes of epithelial cells. To model the possible induction of release of intracellular virus by interaction of PBMC with mucosal epithelial cells from their BL membranes, we cocultivated the activated and nonactivated PBMC from the BL membranes of polarized cells containing HIV-1 at day 6 with or without ionomycin as shown in S3A Fig. After 4 h cocultivation, culture medium from AP and BL membranes was collected separately for ELISA p24. The data showed activated PBMC induced viral release from both AP and BL membranes with or without ionomycin treatment (Fig 5C). Notably, AP release of virus was not due to direct interaction of PBMC, because epithelial–lymphocyte cocultivation was performed from BL membranes of polarized cells. Nonactivated PBMC did not induce viral release in the absence of ionomycin.

During inflammation of mucosal epithelium, lymphocytes may interact with mucosal epithelial cells from both AP and BL membranes. We thus cocultivated activated PBMC and epithelial cells from AP or BL membranes (S3A Fig). The interaction of PBMC with epithelial cells induced the release of virus from both membranes (Fig 5D); however, BL release was significantly higher than AP release. These data clearly demonstrate that interaction of PBMC with AP membranes induces release of virus from BL membranes and vice versa.

In Transwell inserts with a 3-μm pore size, lymphocytes from the opposite side of the inserts will interact with epithelial cells via the pores [79]. To confirm this, we compared PBMC–BL membrane cocultivation using Transwell inserts with 0.42- or 3-μm pore size (Fig 5E). Induction of HIV-1 release occurred only when inserts with a 3-μm pore size were used (Fig 5E, upper panel). The pore size of 0.42 μm is too small to allow the interaction of lymphocytes with epithelium [80]. A decrease in TER also occurred only during cocultivation of PBMC–BL membranes with inserts of 3-μm pore size (Fig 5E, lower panel). These data clearly show that direct interaction of PBMC with epithelial cells through inserts with a 3-μm pore size is critical for the induction of HIV-1 release. Nuclear staining of polarized epithelial cells without cocultivation with lymphocytes showed that epithelial cells did not transmigrate via 3-μm-pore-size Transwell inserts (S3B Fig) due to their larger size (~15–60 μm) [81, 82].

To determine if HIV-1 spread may occur from tonsil epithelium into CD4+ T lymphocytes from the same individual, we propagated matching tonsil keratinocytes and activated CD4+ T lymphocytes from two subjects. HIV-1_SF33_ was sequestered in polarized tonsil epithelial cells for 6 days. Examination of one set of polarized cells by ELISA p24 showed that virus was sequestered in the tonsil epithelial cells (Fig 5F, upper panel). The autologous CD4+ T lymphocytes were added to the AP surface of matching epithelial cells. After 4 h, lymphocytes were collected and grown for 12 days, which showed HIV-1 infection (Fig 5F, lower panel).

### Tumor Necrosis Factor-α (TNF-α) and Interferon-γ (IFN-γ) induce the release of sequestered HIV-1 from tonsil epithelial cells

**Fig 6:**
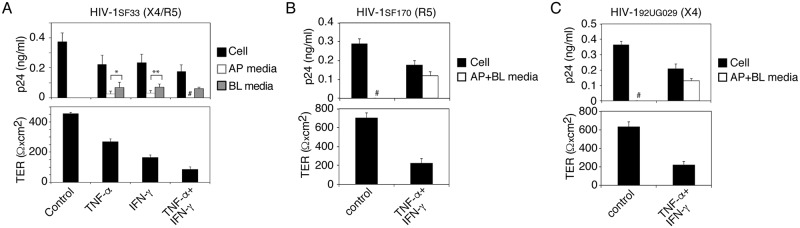
Proinflammatory cytokines TNF-α and IFN-γ induce release of sequestered HIV-1 from tonsil epithelial cells. (A) Polarized tonsil cells sequestering HIV-1_SF33_ for 6 days were treated with recombinant TNF-α and IFN-γ alone and in combination for 24 h. TER of polarized cells was then measured (lower panel), and cells and AP and BL medium were tested for HIV-1 p24 (upper panel). (B, C) Polarized tonsil epithelial cells containing sequestered R5-tropic HIV-1_SF170_ and X4-tropic HIV-1_92UG029_ at day 6 were treated with TNF-α and IFN-γ in combination for 24 h. Then, intracellular HIV-1 and released virions combined from AP and BL medium were examined by ELISA p24 (upper panels) and TER was measured (lower panels). Data are shown as mean ± SEM of three independent experiments, each in triplicate (n = 3).*P < 0.01, **P < 0.03, BL release of virus compared with AP release. # Not detected.

Activated PBMC secrete multiple proinflammatory cytokines, including TNF-α and IFN-γ [83], which reorganize the actin cytoskeleton, leading to the depolarization of epithelial cells [84–86]. To examine the role of TNF-α and IFN-γ in the release of intracellular HIV-1 from polarized cells, we treated cells with recombinant TNF-α and IFN-γ alone and in combination. After 24 h, TER was measured, and culture medium from AP and BL membranes and cells was tested separately for HIV-1. Both cytokines induced the depolarization of epithelial cells and the release of intracellular virions. Release of virus was correlated with reduction of intracellular virions (Fig 6A, upper panel). TER was reduced more with the combination of TNF-α and IFN-γ than with either alone (Fig 6A, lower panel). Virus was released from both AP and BL surfaces of polarized cells treated with TNF-α or IFN-γ alone, but virus was detected only from BL medium after combined TNF-α and IFN-γ treatment. This could be due to paracellular leakage of virus from the upper to the lower chambers of inserts. To test this possibility, in parallel experiments we examined TNF-α- and/or IFN-γ- treated and untreated cells for TER, paracellular permeability and cell viability. As a control, one set of cells were treated with 10 mM EDTA to induce paracellular permeability. Analysis showed that both TNF-α and IFN-γ reduced TER by ~40 and 50%, respectively (S4 Fig, upper panel). However, TER was substantially reduced (~70%) by the combination of TNF-α and IFN-γ. Paracellular leakage was detected only in cells treated with the combination of TNF-α and IFN-γ (S4 Fig, middle panel); this was consistent with the substantial reduction of TER. These data confirmed that lack of AP release of HIV-1_SF33_ from tonsil cells treated with the combination of TNF-α and IFN-γ was due to paracellular leakage of virus. Analysis of cell viability showed that TNF-α and IFN-γ treatment alone and in combination did not have a toxic effect on polarized tonsil epithelial cells (S4 Fig, lower panel).

To determine if TNF-α and IFN-γ induce release of X4- and R5-tropic HIV-1, we treated polarized tonsil epithelial cells containing R5-tropic HIV-1_SF170_ and X4-tropic HIV-1_92UG029_ viruses with TNF-α and IFN-γ in combination for 24 h. Analysis of virus release in combined AP and BL medium showed that TNF-α and IFN-γ induced the release of both R5- (Fig 6B, upper panel) and X4- (Fig 6C, upper panel) tropic viruses, and that this release was dependent on a reduction in TER (Fig 6B and 6C, lower panels).

### Disruption of the cortical actin cytoskeleton is critical for the release of sequestered HIV-1 from polarized epithelial cells

**Fig 7:**
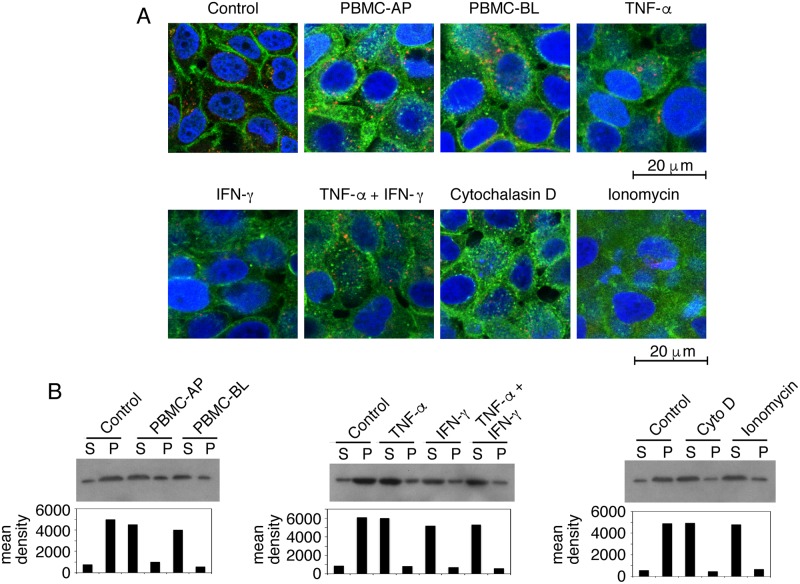
Disruption of cortical actin cytoskeleton is critical for HIV-1 release. (A) Polarized tonsil epithelial cells containing intracellular HIV-1_SF33_ for 6 days were incubated with activated PBMC for 4 h from AP or BL surfaces of polarized cells. Then PBMC were removed and epithelial cells were fixed. In parallel experiments, polarized cells were treated with TNF-α and/or IFN-γ for 24 h, and ionomycin and cytochalasin D for 30 min. Cells without PBMC coculture and not treated served as a control. Cells were then fixed and costained for fluorescence-labeled phalloidin (green) and for HIV-1 p24 (red). Similar results were obtained in 5 independent experiments. (B) Experiments were performed by cocultivation of tonsil cells containing HIV-1_SF33_ with PBMC, or treated with cytokines, ionomycin and cytochalasin D as described above (A). Then cells were lysed and subjected to ultracentrifugation. Supernatant (S) containing G-actin and pellet (P) containing F-actin were detected by Western blot. A representative Western blot was selected from two independent experiments. The mean density of the protein bands is shown under the blot.

To examine the status of cortical actin in HIV-1 released epithelial cells, we cocultured polarized tonsil epithelial cells containing HIV-1_SF33_ with activated PBMC from AP or BL membranes for 4 h. In parallel experiments, we treated tonsil cells with ionomycin and cytochalasin D for 30 min, and with TNF-α or IFN-γ for 24 h. The cells were then costained for HIV-1 and fluorescein isothiocyanate (FITC)-labeled phalloidin, which binds to filamentous (F)-actin, the main component of the actin cortex [47, 87]. Confocal microscopy showed that F-actin localization in control cells was in a ring shape (Fig 7A), indicating localization of F-actin in the cortex beneath the plasma membranes. In contrast, the F-actin staining pattern in tonsil epithelial cells cocultivated with PBMC as well as epithelial cells treated with TNF-α, IFN-γ, ionomycin, and cytochalasin D was a discontinuous cytoplasmic patch, indicating the disruption and reorganization of cortical actin. HIV-1 staining in these epithelial cells was in a vesicular pattern consistent with viral localization within the endosomal compartments.

Disruption/reorganization of cortical actin was also examined by separation of cortical F-actin from globular (G)-actin. Experiments were performed as described above, cells were lysed, and F-actin was sedimented by ultracentrifugation [88, 89]. Western blot analysis of supernatant and pellet, which contain G- actin and F-actin, respectively, showed that untreated (control) tonsil epithelial cells contained ~85% F actin in the pellet and ~15% G actin in the supernatant (Fig 7B). These data indicate that in the untreated epithelial cells the majority of F-actin is associated with the cortical actin network. In contrast, in the tonsil cells cocultivated with PBMC from AP or BL membranes, F-actin was 20–30% in the pellet, and G-actin was 70–80% in the supernatant. A similar trend was seen in the TNF-α−, IFN-γ−, ionomycin-, and cytochalasin D-treated cells, indicating a reduction of F-actin in the pellet (cortex) and an increase of G-actin in the soluble fraction.

Together, these data indicate that disruption/reorganization of the cortical actin cytoskeleton is critical for the release of intravesicular HIV-1.

### Adhesion of PBMC to epithelial cells via LFA-1 and ICAM-1 interaction and the spread of sequestered HIV-1 from epithelia to lymphocytes

**Fig 8:**
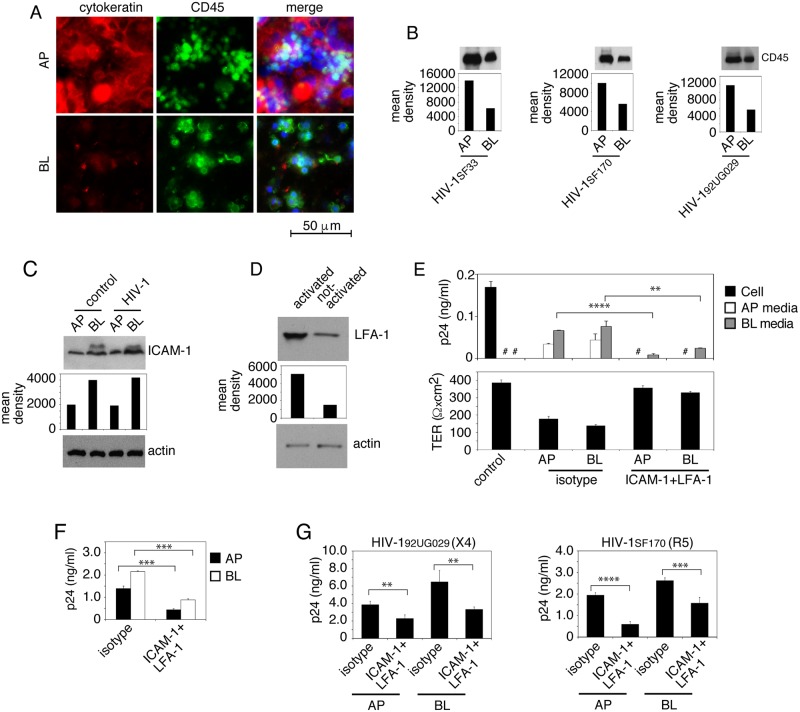
Release and spread of sequestered HIV-1 from epithelial cells to PBMC through interaction of ICAM-1 of tonsil epithelial cells and LFA-1 of lymphocytes. (A) Activated PBMC were added to AP or BL membranes of polarized tonsil epithelial cells containing HIV-1_SF33_ for 6 days, and after 4 h cocultured cells were fixed and immunostained for CD45 (green) and cytokeratin (red). Nuclei are counterstained with TO-PRO (blue). (B) Activated PBMC were added to AP and BL membranes of polarized tonsil cells containing HIV-1_SF33_, HIV-1_SF170_, or HIV-1_92UG029_ for 6 days. After 4 h incubation, PBMC were collected from AP and BL chambers separately and used for detection of CD45 by Western blotting. (C) ICAM-1 was detected in AP and BL membranes of polarized tonsil cells with or without intracellular HIV-1_SF33_ by domain-specific biotinylation assay. (D) LFA-1 was detected in activated and nonactivated PBMC by Western blot. (B, C and D) The mean density of CD45, ICAM-1 and LFA-1 protein bands is shown under the blot. The presence of β-actin ensured equal loading. Immunoblots were performed at least twice and a representative figure is shown. (E, F) Polarized tonsil cells containing intracellular HIV-1_SF33_ and activated PBMC were preincubated with anti-ICAM-1 and LFA-1 antibodies, respectively, or with isotype controls. Epithelial cells and lymphocytes were cocultivated for 4 h from the AP or BL membranes of polarized cells. Epithelial cells that were not cocultivated with PBMC served as a control. TER was measured (E, lower panel). Culture medium was examined for released virus (E, upper panel). PBMC were grown for 7 days and examined by ELISA p24 (F). Data represent one of two independent experiments and are shown as mean ± SEM of triplicate values. ***P < 0.0001, ****P < 0.00004, compared with the control isotype antibodies. # Not detected. (G) Polarized tonsil epithelial cells containing X4-tropic HIV-1_92UG029_ or R5-tropic HIV-1_SF170_ were cocultured with CD4+ T lymphocytes from AP or BL membranes of epithelial cells in the presence or absence of antibodies against ICAM-1 and LFA-1. After 4 h lymphocytes were collected and grown for 4 days (X4-tropic HIV-1_92UG029_) or 7 days (R5-tropic HIV-1_SF170_). Lymphocytes were then examined for HIV-1 infection by ELISA p24. Data are shown as mean ± SEM of three independent experiments (in triplicate) (n = 3). **P<0.001, and ***P<0.0007, **** P<0.00005.

To determine if PBMC adhesion to epithelial cells containing HIV-1 is critical for release and spread of sequestered virions from epithelial cells to lymphocytes, we cocultivated polarized tonsil epithelial cells containing dual-tropic HIV-1_SF33_, R5-tropic HIV-1_SF170_ and X4-tropic HIV-1_92UG029_ viruses at day 6 of sequestration with activated PBMC from AP or BL membranes of epithelial cells. After 4 h, one set of cocultivated cells were coimmunostained for the epithelial marker pan cytokeratin and the lymphocyte marker CD45. Results showed that lymphocytes were attached to the AP and BL membranes of epithelial cells (Fig 8A). In another set of inserts, PBMC were collected from the epithelial cell surface and examined for CD45 expression by Western blot assay. Data revealed that the number of PBMC collected from the BL surface was about half that of PBMC collected from the AP surface (Fig 8B).

Analysis of ICAM-1 expression in polarized tonsil epithelial cells by a domain-specific labeling assay showed that ICAM-1 expression was detected from both AP and BL surfaces; however, its expression in BL membranes was approximately twofold higher than that in AP membranes (Fig 8C). HIV-1 sequestration in tonsil cells did not increase the basal level of polarized expression of ICAM-1, indicating that intravesicular viral sequestration may not play a role in the induction of ICAM-1 expression.

Western blot analysis of LFA-1 expression in activated and nonactivated PBMC showed that its expression in activated PBMC was approximately 3-fold higher than that in nonactivated PBMC (Fig 8D).

To identify the role of ICAM-1 and LFA-1 in epithelial–lymphocyte interaction and in HIV-1 release and spread, the ICAM-1–LFA-1 interaction in cocultivated cells was inhibited by specific antibodies against these proteins. Analysis of HIV-1_SF33_ release showed that inhibition of ICAM-1 and LFA-1 interactions from AP and BL surfaces led to complete inhibition of virus release from the AP membrane of epithelial cells (Fig 8E, upper panel). HIV-1 release from BL medium was reduced by ~60–80%. Analysis of TER showed that inhibition of LFA-1/ICAM-1 interaction by antibodies did not reduce the TER of polarized cells, indicating that PBMC binding to tonsil cells via LFA-1-ICAM-1 is important in depolarization of epithelial cells and induction of viral release (Fig 8E, lower panel).

Inhibition of the LFA-1–ICAM-1 interaction also substantially (~65–70%) reduced virus spread from both AP and BL surfaces of epithelial cells to activated PBMC (Fig 8F).

Analysis of the role of ICAM-1–LFA-1 in the spread of R5- and X4-tropic HIV-1 from tonsil epithelial cells to CD4+ T lymphocytes showed that antibodies against ICAM-1 and LFA-1 significantly reduced the spread of both R5- and X4-tropic HIV-1 viruses to CD4+ lymphocytes compared to isotype antibodies (Fig 8G). Detection of R5-tropic virus infection in CD4+ T lymphocytes was lower than that of X4-tropic virus infection, which should be due to a lower level of CCR5 expression than CXCR4 expression in CD4+ T lymphocytes.

**Fig 9:**
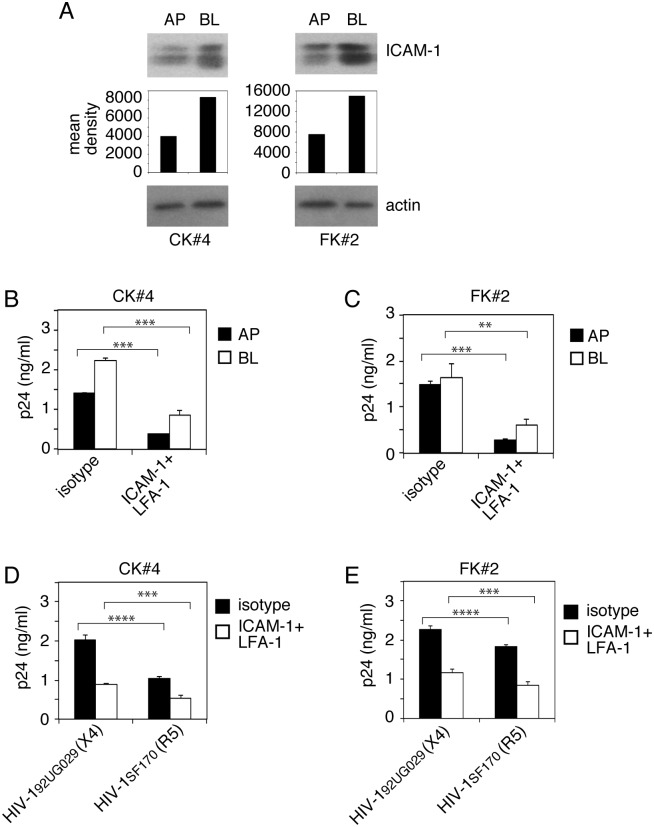
Spread of sequestered HIV-1 from cervical and foreskin epithelial cells to lymphocytes. (A) Cell surface expression of ICAM-1 was examined in AP and BL membranes of polarized CK#4 and FK#2 cells by domain-specific biotinylation assay. The mean density of ICAM-1 protein bands is shown under the blot. β-Actin was detected to confirm equal loading. Experiments were performed at least twice and a representative figure is shown. (B, C) Polarized cervical CK#4 (B) and foreskin FK#2 (C) cells containing intracellular HIV-1_SF33_ and activated PBMC were preincubated with antibodies against ICAM-1 and LFA-1 or with their isotype controls, respectively. Then, epithelial cells and PBMC were cocultured for 4 h. PBMC were collected, grown for 4 days, and examined by ELISA p24. (D, E) Polarized cervical and foreskin epithelial cells sequestering X4-tropic HIV-1_92UG029_ and R5-tropic HIV-1_SF170_ viruses were cocultured with CD4+ T lymphocytes in the presence of absence of ICAM-1 and LFA-1 antibodies or their isotype controls. Lymphocytes were collected and grown for 4 days (HIV-1_92UG029_) or 7 days (HIV-1_SF170_), and infection was examined by ELISA p24. (B-E) Data are shown as mean ± SEM of three independent experiments, each in triplicate (n = 3). **P < 0.001, ***P < 0.0001, ****P < 0.00001, compared with the control isotype antibodies.

We also examined the role of ICAM-1 and LFA-1 interaction in HIV-1 spread from cervical and foreskin epithelial cells to lymphocytes. Domain-specific labeling of ICAM-1 expression in AP and BL membranes of cervical and foreskin cells showed that ICAM-1 was expressed in both surfaces of these cells, although BL expression was higher (Fig 9A). Cocultivation of activated PBMC with polarized cervical and foreskin epithelial cells containing dual-tropic HIV-1_SF33_ in the presence of antibodies to LFA-1 and ICAM-1 reduced viral spread from AP and BL membranes of epithelial cells by 40–50% (Fig 9B and 9C). Similarly, these antibodies reduced the spread of R5-tropic HIV-1_SF170_ and X4-tropic HIV-1_92UG029_ virus to CD4+ T lymphocytes by ~70% and 50%, respectively (Fig 9D and 9E).

### Inhibition of MVB and vacuole formation reduced HIV-1 sequestration in epithelial cells, and virus spread from epithelia to lymphocytes

**Fig 10:**
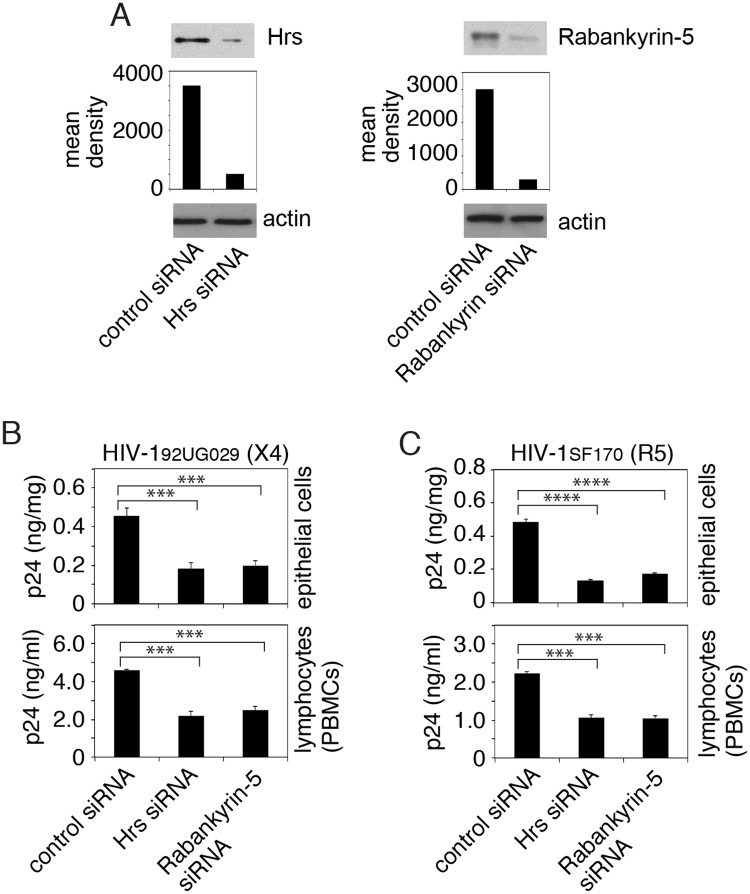
Inhibition of MVB and vacuole formation reduced HIV-1 sequestration and virus spread to lymphocytes. (A) Polarized tonsil cells were transfected with control siRNA or siRNAs against Hrs and rabankyrin-5. After 72 h, expression of Hrs and rabankyrin-5 was examined by Western blot. The mean density of protein bands is shown under the blot. β-Actin was detected to confirm equal loading. Immunoblots were performed at least twice and a representative figure is shown. (B, C) Tonsil epithelial cells transfected with control siRNA or siRNAs specific to Hrs or rabankyrin-5 at 72 h after transfection were exposed to HIV-1_92UG029_ or HIV-1_SF170_. After 3 days, one set of siRNA-transfected cells were examined for intracellular HIV-1 (upper panels). The next set of siRNA-transfected cells were cocultured with activated CD4+ T lymphocytes (lower panels). Four hours later, lymphocytes were collected and grown for 4 days for HIV-1_92UG029_ and 10 days for HIV-1_SF170_. HIV-1 infection was examined by ELISA p24. Data represent one of three independent experiments and are shown as mean ± SEM of triplicate values. ***P < 0.0001 and ****P < 0.00001, compared with the control siRNAs.

The experiments described above showed that sequestered HIV-1 colocalized with markers of MVBs and vacuoles (Fig 3). To examine the role of MVBs and vacuoles in HIV-1 sequestration in epithelial cells and in the spread of virus from epithelial cells to lymphocytes, we transfected tonsil epithelial cells with siRNAs against Hrs and rabankyrin-5, which are critical proteins for MVB and vacuole formation, respectively [21, 70, 90]. After 72 h, one set of cells were used for detection of Hrs and rabankyrin-5 expression by Western blot, which showed that siRNA transfection reduced Hrs and rabankyrin by ~80% and 90%, respectively (Fig 10A).

The next set of siRNA-transfected tonsil cells were used for sequestration of X4-tropic HIV-1_92UG029_ and R5-tropic HIV-1_SF170_ viruses. Three days later, one set of cells were examined for intracellular HIV-1. Data showed that both Hrs and rabankyrin siRNAs reduced sequestration of both X4- and R5-tropic viruses in the tonsil epithelial cells by ~60% and 70%, respectively (Fig 10B and 10C, upper panels).

The last set of siRNA-transfected tonsil epithelial cells containing virus were cocultured with activated CD4+ T lymphocytes for 4 h (Fig 10B and 10C, lower panels). Analysis of HIV-1 infection of CD4+ T lymphocytes showed that transfection of Hrs and rabankyrin-5 siRNAs reduced the spread of both X4-tropic HIV-1_92UG029_ and R5-tropic HIV-1_SF170_ viruses from tonsil epithelial cells to CD4+ T lymphocytes by ~55–60% (Fig 10B and 10C, lower panels). Similar experiments were performed with dual-tropic HIV-1_SF33_ virus using CD4+ T lymphocytes from PBMC and tonsil tissues. Data showed that inhibition of Hrs and rabankyrin expression in epithelial cells reduced viral sequestration in epithelial cells and its spread from epithelial cells to CD4+ T lymphocytes isolated from PBMC and tonsil tissues (S5 Fig). Thus, the inhibition of MVB and vacuole formation reduced virus sequestration in epithelial cells and its spread from epithelial cells to CD4+ T lymphocytes, indicating the critical role of intravesicular viral sequestration in HIV-1 mucosal transmission.

## Discussion

**Fig 11:**
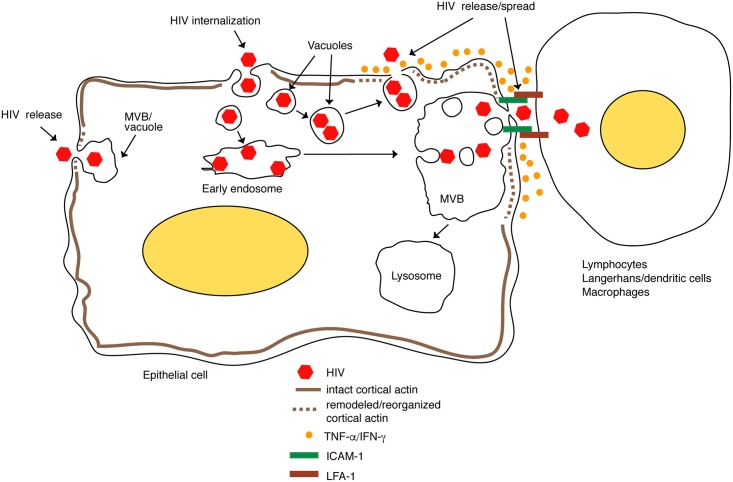
Model of release of HIV-1 sequestered in mucosal epithelium. HIV-1 penetrates the endosomal compartments of mucosal epithelium and is sequestered in the MVB and vacuoles for several days without release. The activated lymphocytes, macrophages and dendritic cells infiltrating the mucosal epithelium bind to epithelial cells through LFA-1 of lymphocytes and ICAM-1 of epithelial cells, inducing disruption of the cortical actin cytoskeleton of epithelial cells and leading to the release of intravesicular virions. Furthermore, activated immune cells secrete TNF-α and IFN-γ, which may disrupt/reorganize the cortical actin that induces virus release. Adhesion of immune cells to epithelial cells that sequester HIV-1 through LFA-1/ICAM-1 interaction may facilitate HIV-1 spread from epithelial to immune cells through the virologic synapse.

We have shown that HIV-1 penetrates polarized tonsil, cervical and foreskin epithelial cells. Most internalized virions are sequestered in the MVB and vacuoles of epithelial cells for several days without release but with preservation of infectious activity (Fig 11). The interaction of activated PBMC and CD4+ T lymphocytes with epithelial cells containing virus induces the spread of sequestered intravesicular virions to lymphocytes.

HIV-1 intraepithelial sequestration without HIV-1 release was common in tonsil, cervical and foreskin epithelial cells isolated from independent donors, suggesting that this phenomenon may be relevant to the biological functions of both oral and genital mucosal epithelia. Although these epithelia are found at different anatomical sites, they have similar morphostructural features—squamous epithelial morphology and stratified organization—and serve as portals of entry for HIV-1 [1–6]. They may have similar mechanisms for HIV-1 sequestration in their endosomal/vesicular compartments and may play a critical role in viral transmission. Although HIV-1 sequestration has been observed in cervical, prostate and kidney epithelial cells [32, 91, 92], intracellular compartments that retain virions have not been fully characterized, and the molecular mechanisms of viral sequestration have not been investigated.

HIV-1 release from polarized epithelial cells by ionomycin indicates that viral exocytosis from MVB and/or vacuoles is inducible and may be regulated by extracellular stimuli. Depolarization of epithelial cells and disruption of cortical actin after ionomycin, cytochalasin D or cytokine treatment and lymphocyte cocultivation show that disruption of the cortical actin cytoskeleton beneath the membrane is critical for the release of sequestered virions. Disruption of a pre-existing cortical actin network by elevation of Ca^2++^and/or cytochalasin D treatment is required for the fusion of endosomal membranes with plasma membrane, leading to induction of exocytosis [75, 93].

Ionomycin treatment of productively HIV-1-infected epithelial 293-T cells and MOLT T lymphocytes did not change viral infection but increased virus release [34]. Intracellular HIV-1 virions were detected in the late endosomes/MVBs and vacuoles, suggesting that during productive infection some virions may be sequestered in the MVBs/vacuoles and released by ionomycin-induced increase of intracellular calcium [34].

Induction of cell-to-cell spread of HIV-1 from epithelial cells to activated PBMC and CD4+ T lymphocytes by direct interaction through LFA-1/ICAM-1 could be due to the formation of virological synapses between these cells. The higher level of HIV-1 spread from BL membranes than from AP membranes is consistent with the higher level of ICAM-1 expression in BL membranes. It is also consistent with the physiology of epithelia–lymphocyte interaction in the mucosal epithelium; i.e., under physiological and inflammatory conditions migratory lymphocytes from lamina propria initially may interact with BL membranes of epithelial cells. Interaction of nonactivated lymphocytes with epithelial cells did not lead to virus release, although nonactivated lymphocytes expressed detectable LFA-1. This suggests that activated lymphocytes in addition to LFA-1 may express other cell surface adhesion proteins, which also may interact with epithelial cells and play a role in the induction of virus release.

The interaction of LFA-1 and ICAM-1 in the immunologic synapse increases intracellular calcium and reorganizes actin cytoskeleton, including the cortical actin network [94–96]. The interaction of lymphocytes and endothelial cells via LFA-1/ICAM-1 binding also increases intracellular calcium and induces the depolarization of endothelial cells and reorganization of the actin network [97, 98]. In our epithelia–lymphocyte cocultivation model, the ICAM-1/LFA-1-induced depolarization of epithelial cells and reorganization of the actin network was critical for (i) viral exocytosis from epithelial cells and (ii) cell-to-cell viral spread from epithelial cells to lymphocytes. Both pathways may be simultaneously regulated and induced by the LFA-1/ICAM-1–activated increase in intracellular calcium, which may trigger remodeling of actin cytoskeleton and depolarization of epithelial cells.

Epithelia–lymphocyte interaction also induces the release of virus from opposite membranes, which do not have direct contact with the lymphocytes. This suggested that LFA-1/ICAM-1–induced disruption/reorganization of cortical actin may occur globally. It has been shown that cell-associated HIV-1 transcytosis via polarized epithelia is 10- to 100-fold higher than that of cell-free viral transcytosis [1, 6, 99, 100]. This could be due to LFA-1/ICAM-1–mediated interaction of HIV-infected lymphocytes with the AP surface of epithelial cells, which may in turn disrupt/reorganize the cortical actin cytoskeleton of epithelial cells, leading to a substantial increase in viral penetration from AP membranes and the virus’s subsequent release from BL membranes of epithelial cells, i.e., facilitated viral transcytosis.

Induction of epithelial depolarization, disruption of cortical actin, and HIV-1 release by recombinant TNF-α and/or IFN-γ indicates that activated immune cells may also have a paracrine effect on the release of sequestered virus from epithelial cells by PBMC-secreted proinflammatory cytokines. Proinflammatory cytokines, including TNF-α and IFN-γ, induce myosin light chain phosphorylation through activation of NF-κB and RhoA signaling pathways [101–107]. This will lead to reorganization/remodeling of cortical actin and disruption of cell junctions, resulting in depolarization of epithelial cells [101–106, 108–114]. Thus, inflammation in the mucosal epithelium by other viral and bacterial infection may play a critical role in the release of intraepithelial HIV-1, increasing the risk of viral mucosal transmission.

Reduction of HIV-1 sequestration and its spread from epithelial cells to lymphocytes by siRNA-mediated inhibition of Hrs and rabankyrin-5 expression in epithelial cells shows the critical role of virus-containing MVBs and vacuoles in HIV-1 mucosal transmission. Male circumcision may reduce HIV-1 transmission by up to 70% [115–117], suggesting that intravesicular HIV-1 sequestration in foreskin epithelium in vivo may occur, and therefore removal of epithelium prevents the spread of sequestered virus.

We have shown that human beta defensin-2 (hBD2) and -3 cointernalize with HIV-1 into vesicles of infant tonsil epithelium and inactivate virus in the endosomes [118]. However, expression of hBD2 and -3 expression varies between different epithelia. hBD2 and -3 were highly expressed in adult oral mucosal epithelia, in contrast to infant oral and adult cervical and foreskin epithelia, which express these innate proteins at low levels [1, 118, 119].

In summary, we have shown that infectious HIV-1 is sequestered in the endosomes of tonsil, cervical and foreskin epithelial cells. The interaction of activated PBMC and CD4+ T lymphocytes with these epithelia through lymphocyte LFA-1 and epithelial ICAM-1 receptors induced the release and spread of intraepithelially trapped HIV-1 to lymphocytes. Development of therapeutic approaches to eliminate vesicles containing HIV-1 and/or to inactivate intravesicular virions in mucosal epithelium may help to prevent HIV-1 MTCT via infant tonsil epithelium as well as viral transmission through adult cervical and foreskin mucosa.

## Materials and methods

### Ethics statement

This study was conducted according to the principles expressed in the Declaration of Helsinki and was approved by the Committee on Human Research of the University of California–San Francisco (IRB approval # H8597-30664-03). All human subjects provided written informed consent for the collection of tissue samples. The parents provided informed consent for all minors.

### Viruses and cells

Laboratory-adapted dual (X4-R5)-tropic HIV-1_SF33_ and the primary isolates R5-tropic HIV-1_SF170_ and X4-tropic HIV-1_92UG029_ were grown in PBMC activated with 2.5 μg/ml phytohemagglutinin (Sigma) and 1 μg/ml interleukin-2 (BD Biosciences) for 3 days. Viral stocks were titered by p24 concentration using HIV-1 p24 ELISA (ELISA p24) (PerkinElmer) according to the manufacturer’s instructions.

TZM-bl cells (NIH AIDS Research and Reference Reagent Program) expressing HIV-1 receptors/coreceptors CD4, CXCR4, and CCR5, were contributed by John Kappes and Xiaoyun Wu [120, 121].

Primary tonsil epithelial keratinocytes were established from tonsil tissue from 15 HIV-negative children <5 years of age after routine tonsillectomy. Primary cervical keratinocytes were established from ectocervical tissue specimens from 4 HIV-negative donors. Foreskin keratinocytes from two independent donors were obtained from Lonza. The keratinocytes were grown in keratinocyte growth medium (KGM gold) (Lonza). Keratinocytes were used at early passages and frozen in liquid nitrogen.

Polarized epithelial cells were established in 12-well Transwell two-chamber permeable filter inserts with 0.4-μm or 3-μm pore size, as described in our previous work [1, 16, 17, 122]. The transepithelial electrical resistance (TER) of polarized cells was measured using a Millicell-ERS voltohmmeter (Millipore). Paracellular permeability of polarized cells was evaluated by adding horseradish peroxidase-conjugated goat anti-mouse IgG (Fab’)^2^ (Jackson ImmunoResearch) to the upper compartments of Transwell inserts and, after 30 min, photometrically assaying horseradish peroxidase in the medium from the lower compartment using *o*-phenylenediamine dihydrochloride as the substrate [123]. To induce paracellular leakage of polarized cells, 10 mM EDTA with IgG was added to the AP medium. The values are expressed as optical density (OD, 450 nm).

### Assessing HIV-1 transcytosis and sequestration in polarized epithelium

HIV-1 at 20 ng/ml of p24 was added to the AP surface of polarized epithelial cells, and 4 h later uninternalized virions were removed with 0.05% trypsin for ~3–4 min at room temperature [12, 118]. Trypsin was immediately inactivated with keratinocyte growth medium containing 10% fetal bovine serum (FBS) (HyClone), and cells were washed and maintained for 1, 3, 6 and 9 days. The culture medium from lower chambers was collected for detection of transcytosed virions [1]. Cells were dissociated with 0.25% trypsin at 37°C and used for detection of intracellular virus. Transcytosed and intracellular virus was detected by HIV-1 p24 ELISA. Each experiment was performed 2 to 6 times using cells from independent donors with at least triplicate Transwell inserts for each experimental condition.

### Release of sequestered HIV-1 from polarized epithelial cells by ionomycin, cytochalasin D, TNF-α and IFN-γ treatment and cocultivation of epithelial cells with PBMC and CD4+ T lymphocytes

All experiments for HIV release and spread were performed using Transwell inserts with 3-μm pore size. To induce HIV-1 release from epithelial cells, polarized cells containing intracellular virions were washed and treated with 10 μM calcium ionophore ionomycin and/or 12 μg/ml cytochalasin D (both from Sigma), which was dissolved in dimethyl sulfoxide. Culture medium in these experiments contained 2 mM calcium chloride. Untreated control cells and ionomycin- and/or cytochalasin D-treated cells were incubated with a similar concentration of dimethyl sulfoxide. Culture medium was collected separately from AP and BL chambers of the Transwell inserts and used for detection of released virions by ELISA p24.

PBMC were isolated from heparinized blood using a Ficoll-Paque Plus density gradient (Sigma). CD4+ T lymphocytes were then isolated by positive selection using anti-CD4 microbeads (Miltenyi Biotec) [2]. CD4+ T lymphocytes were also purified from tonsil tissues [124]. The purity of CD4+ T lymphocytes—~98%—was verified by flow cytometry. PBMC and CD4+ T lymphocytes were activated with 2.5 μg/ml phytohemagglutinin (Sigma) and 1 μg/ml interleukin-2 (BD Biosciences) for 3 days. For cocultivation of activated or nonactivated PBMC or purified CD4+ T lymphoctyes with polarized epithelial cells containing HIV, ~10^6^ lymphocytes were washed twice and added to AP or BL surfaces of epithelial cells. The ratio of lymphocytes to epithelial cells was ~2:1.

For lymphocyte cocultivation with the AP surface of epithelial cells, lymphocytes were added to the upper chambers of Transwell filter inserts, where the keratinocytes were grown with the AP cell surfaces facing up. Under this condition, lymphocytes have direct contact with the AP surfaces of epithelial cells. For lymphocyte cocultivation with the BL surface of epithelial cells, keratinocytes were grown on the lower surfaces of filter inserts, with the AP cell surfaces facing the lower chamber and the BL cell surfaces attached to the lower face of the filter inserts [17, 125]. Filter inserts were placed upside down in petri dishes, and cells in 50 μl of medium were added to the filter surface. Inserts with cells were incubated for 4–8 h at 37°C in 5% CO_2_ to allow attachment of cells to the filters. Filter inserts were then placed in 12-well plates in the correct orientation. Attached keratinocytes formed polarized monolayers. For HIV-1 penetration, virus was added to the lower chambers of the inserts, which were placed on a shaker at slow motion for 1 h at 37°C. This procedure leads to entry of virus from the lower chamber onto the AP surface of polarized cells. Adding lymphocytes to the upper chambers of the inserts permitted binding of lymphocytes to the BL surface of polarized cells. After 4 h, medium and lymphocytes were collected by pipeting and centrifuged for 10 min at 1,200 rpm. Supernatant was used for analysis of released virions. PBMC were grown for 3–12 days and analyzed for HIV-1 infection. Release of virus and infection of PBMC were examined by ELISA p24.

For inhibition of lymphocyte adhesion to epithelial cells, we incubated polarized epithelial cells with mouse monoclonal antibodies against ICAM-1 (AB20, clone 15.2, 5 μg/ml, Abcam) or isotype controls for 30 min [126]. At the same time, activated PBMC or CD4+ T lymphocytes were incubated with antibodies against the alpha subunit of LFA-1 (CD18a) (AB 3981, clone MEM-83, 2 μg/ml, Abcam) or isotype controls for 30 min [127]. Lymphocytes were added to AP or BL membranes of polarized cells for 4 h. PBMC with culture medium was collected and centrifuged, and supernatant was used to detect released virus. PBMC were cultured for 7 days and examined for HIV-1 infection by ELISA p24.

To induce HIV-1 release by proinflammatory cytokines TNF-α and IFN-γ, we treated cells with recombinant TNF-α and IFN-γ (both from R&D Systems, 10 ng/ml of each). After 24 h, culture medium from AP and BL membranes of polarized cells was collected and examined by ELISA p24.

### Quantification of F-actin

To detect filamentous (F) actin, we lysed polarized tonsil epithelial cells in F-actin stabilization buffer containing 50 mM PIPES (pH 6.9), 50 mM NaCl, 5 mM MgCl_2_, 5 mM EGTA, 5% glycerol, 0.1% Triton X-100, 0.1% NP-40, 0.1% Tween 20, 1 mM ATP, and an EDTA-free cocktail of protease inhibitors (Roche) [128]. The lysate was centrifuged at 100,000 x *g* for 1 h at 37°C. The supernatant (G-actin) and pellets (F-actin) were collected separately and analyzed by Western blot assay using rabbit antibody against actin (Cell Signaling). The protein bands were quantified using Image J, and the mean densities of pixels in the protein bands were measured.

### Immunofluorescence assay

For immunofluorescence assays, polarized epithelial cells were fixed with 4% paraformaldehyde and 2% sucrose in PBS for 5 min, and then permeabilized with 0.01% Triton X-100 in 4% paraformaldehyde for 5 min. For detection of EEA1 and rabankyrin, rabbit antibodies were used (both from Abcam) (1 μg/ml). LAMP1 and LBPA were detected using mouse monoclonal antibodies (Santa Cruz Biotechnology and Millipore, respectively) (1 μg/ml). For detection of HIV-1 p24, we used mouse and rabbit anti-p24 antibodies (NIH AIDS Research and Reference Reagent Program and Abcam) (5 μg/ml of each). For detection of CD45, mouse monoclonal antibodies were used (1.2 μg/ml) (R&D). Secondary antibodies labeled with DyLight 488, DyLight 594 and Alexa Fluor were purchased from Jackson ImmunoResearch. Cell nuclei were counterstained with TO-PRO-3 iodide or DAPI (blue) (Molecular Probes). The specificity of each antibody was confirmed by negative staining with the corresponding primary isotype control antibody. For detection of F-actin, cells were stained with fluorescence-labeled phalloidin (Thermo Fisher Scientific). Cells were analyzed by using a Leica SP5 laser confocal microscope (Leica Microsystems) or Nikon Eclipse E400 fluorescence microscope (Nikon).

### Isolation of vesicular/endosomal compartments containing HIV-1 and vesicular markers

Polarized cells grown in 6-well Transwell inserts (24-mm diameter) were exposed to HIV-1_SF33_ (200 ng/ml) at 37°C for 30 min and 6 days. Uninternalized virions were removed with 0.25% trypsin, and cells were homogenized in a buffer containing 10 mM Tris (pH 7.5), 0.25 M sucrose, 1 mM EDTA, and protease inhibitors [34, 118]. For removal of nuclei, homogenized cells were centrifuged at 1,000 rpm for 10 min at 4°C. The postnuclear supernatant was gently mixed with sucrose to a final concentration of 63%. Samples were placed at the bottom of a centrifuge tube and overlaid with 45% sucrose and then 10% sucrose. Samples were centrifuged at 35,000 rpm for 16 h at 4°C. Fractions were collected from the top, and each fraction was analyzed by Western blotting using antibodies for EEA1, LAMP1, LBPA, rabankyrin, and HIV-1 p24. Because detection of LBPA by conventional Western blot assay is not possible, we used the dot-blot assay as described [129].

### Western blot assay

Cells were extracted with 1.0% Triton X-100 buffer (150 mM NaCl, 10 mM Tris/HCl, pH 8.0, and a cocktail of protease inhibitors). Proteins were separated on an SDS-polyacrylamide gel with a 4–20% gradient. ICAM-1, LFA-1, and CD45 were detected by using mouse and goat antibodies from Abcam and R&D Systems. For detection of Hrs and rabankyrin (Santa Cruz Biotechnology and Abcam, respectively) we used mouse antibodies (1 μg/ml of each). An equal protein load was confirmed by the use of β-actin (Ambio). The protein bands were analyzed for quantification using Image J, and the mean densities of pixels were measured.

### Domain-selective surface labeling assay

Polarized cells were incubated with 200 μg/ml sulfo-NHS-LC-biotin (Thermo Fisher Scientific) from AP or BL membranes for 30 min as described previously [16, 130, 131]. Cells were washed with Tris saline (10 mM Tris-HCl, pH 7.4, 120 mM NaCl) and extracted in 1% Triton X-100 lysis buffer containing protease inhibitors. Biotinylated proteins were precipitated with streptavidin–agarose beads in lysis buffer (Thermo Fisher Scientific). Proteins were separated on a 4–20% Tris-glycine SDS-polyacrylamide gel and transferred to nitrocellulose membranes (GE Healthcare). ICAM-1 was detected by using mouse monoclonal antibody (Abcam). Bands were visualized by using the ECL detection system (GE Healthcare).

### HIV-1 infectivity assay

To determine the infectivity of intraepithelial HIV-1, cells containing virions were trypsinized using 0.25% trypsin [118]. Trypsin was inactivated by culture medium containing 10% fetal bovine serum, and cells were washed with serum-free culture medium. Cells were then homogenized by using motor-driven grinders connected with disposable pellet pestles (Kimble Kontes Pellet Pestle Cordless Motor), which leads to the disruption of vesicles as shown previously [118]. Homogenates were centrifuged at 1000 rpm for 10 min, and supernatants were used to infect TZM-bl cells. HIV-infected TZM-bl cells were maintained for 3 days, and HIV-1 infection was evaluated by detecting luciferase activity in the Bright-Glo Luciferase Assay System (Promega) following the manufacturer’s instructions. The background values of the negative controls were subtracted from signals generated by infectious virus.

### Transfection of cells with small interfering RNAs (siRNAs)

The siRNAs for Hrs and rabankyrin were purchased from Santa Cruz Biotechnology (cat. nos. sc-41232 and sc-93654, respectively). Unrelated (scrambled) siRNAs (sc-37007) were used as controls. Polarized cells were transfected with siRNAs using lipid-based transfection reagents as described [1, 132]. Silencing of genes of interest by siRNAs at 72 h after transfection was confirmed by Western blot assay with antibodies to Hrs and rabankyrin. Protein expression was quantified by measuring the intensity of pixels (mean density) in protein bands using Image J software.

### Statistical analysis

Statistical comparisons were made by a two-tailed Student’s t-test. A p value <0.05 was considered statistically significant. Results are expressed as mean ± SEM.

## Supporting information

S1 Fig Ionomycin and cytochalasin D reduced TER but did not induce the paracellular permeability of polarized tonsil epithelial cells. Polarized tonsil epithelial cells containing HIV-1_SF33_ were treated with ionomycin (10 μM), cytochalasin D (12 μg/ml), or a combination of the two for 30 min. One set of cells were treated with 10 mM EDTA. The TER (upper panel), paracellular permeability (middle panel) and cell viability (bottom panel) were measured in untreated and treated cells. Data are shown as mean ± SEM of three independent experiments, each in triplicate (n = 3).
(TIF)

S2 Fig Cocultivation of activated PBMC with polarized epithelial cells does not induce paracellular permeability of polarized cells. (A) Activated and nonactivated PBMC were added to the AP surface of polarized tonsil epithelial cells, and after 1, 2, 3, 4 and 5 h the paracellular permeability (upper panel) and cell viability (lower panel) were examined. As a control, one set of polarized tonsil cells were treated with 10 mM EDTA for 30 min. (B) Activated PBMC and CD4+ T lymphocytes were added to the AP surface of polarized tonsil epithelial cells at lymphocyte–epithelial ratios of 1:1, 1:2, 1:10 and 1:20. After 4 h TER was measured. (A, B) Data are shown as mean ± SEM of three independent experiments, each in triplicate (n = 3).
(TIF)

S3 Fig Model of cocultivation of PBMC with AP or BL membranes of polarized epithelial cells. (A) To cocultivate PBMC with AP membranes of polarized epithelial cells, cells were grown on the upper surfaces of Transwell filter inserts, with AP membranes facing upward. To cocultivate PBMC with BL membranes of polarized epithelial cells, cells were grown on the lower surfaces of Transwell filter inserts, with AP membranes facing downward. Addition of PBMC to the upper chambers of the Transwell inserts allowed binding of lymphocytes to the AP or BL surfaces of polarized cells. (B) Tonsil epithelial cells were seeded into the upper chamber of Transwell inserts with 0.4-μm (left panel) and 3-μm (right panel) pore sizes. After 12 days cells were fixed and cell nuclei were stained with TO-PRO-3 iodide (blue). Cells were analyzed by confocal microscopy by x-z vertical planes. Similar data were obtained in three independent experiments using tonsil, foreskin and cervical epithelial cells.
(TIF)

S4 Fig Proinflammatory cytokines TNF-α and IFN-γ reduce the TER of polarized tonsil epithelial cells. Polarized tonsil epithelial cells were treated with recombinant TNF-α and IFN-γ alone and in combination for 24 h. Then, untreated (control) and cytokine-treated cells were examined for TER (upper panel), paracellular permeability (middle panel) and cell viability (lower panel). Data are shown as mean ± SEM of three independent experiments, each in triplicate (n = 3).
(TIF)

S5 Fig Inhibition of MVB and vacuole formation reduced HIV-1 sequestration and virus spread to CD4+ T lymphocytes isolated from PBMC and tonsil tissues. Polarized tonsil cells were transfected with control siRNAs or siRNAs against Hrs and rabankyrin-5 and after 72 h were exposed to HIV-1_SF33_. After 3 days, one set of siRNA-transfected cells was examined for intracellular virus (upper panel). The next sets of siRNA-transfected cells were cocultured with activated CD4+ T lymphocytes isolated from PBMC (middle panel) or tonsil tissues (lower panel). Four hours later, lymphocytes were collected and grown for 4 days, and virus infection was examined by ELISA p24. Data are shown as mean ± SEM of three independent experiments, each in triplicate for each experimental condition (n = 3). ***P < 0.0001 and ****P < 0.00001, compared with the control siRNAs.
(TIF)
