# Supplementary figures and images for: Release of HIV-1 sequestered in the vesicles of oral and genital mucosal epithelial cells by epithelial-lymphocyte interaction
Source: PLoS Pathog. 2017 Feb 27;13(2):e1006247. doi: 10.1371/journal.ppat.1006247 (PMC5344537; doi:10.1371/journal.ppat.1006247)

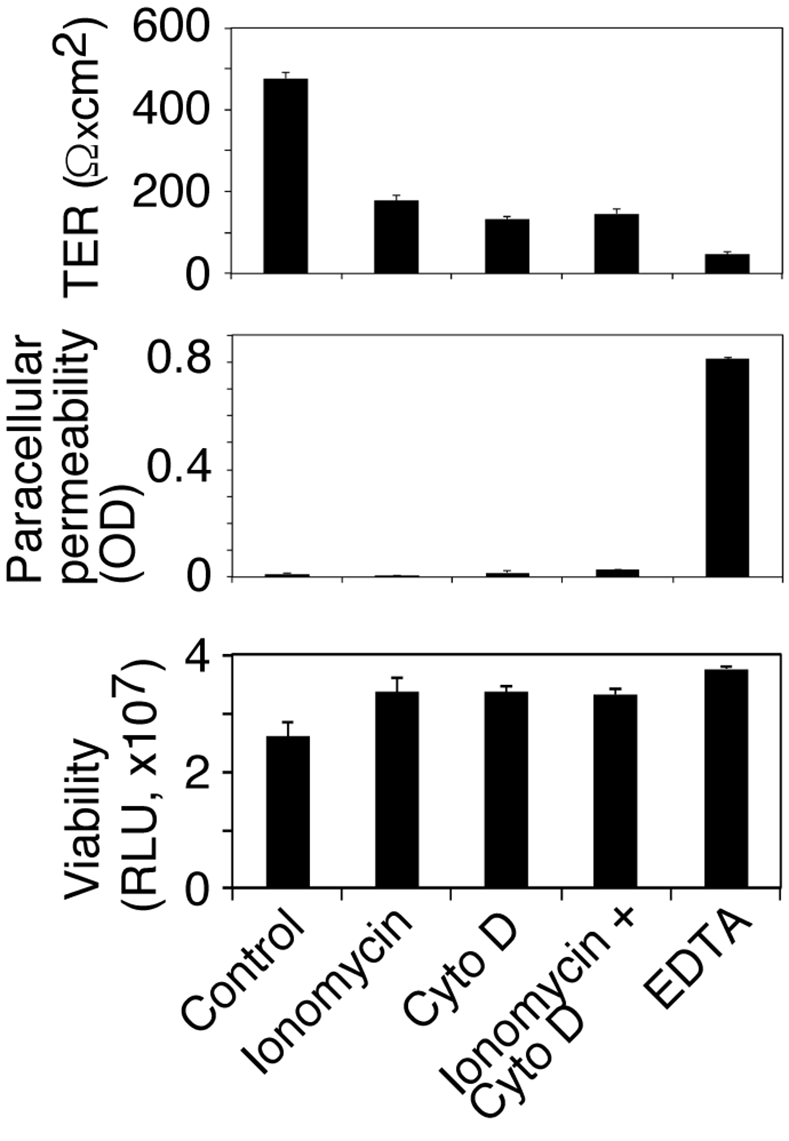

Supplement: S1 Fig — Polarized tonsil epithelial cells containing HIV-1SF33 were treated with ionomycin (10 μM), cytochalasin D (12 μg/ml), or a combination of the two for 30 min. One set of cells were treated with 10 mM EDTA. The TER (upper panel), paracellular permeability (middle panel) and cell viability (bottom panel) were measured in untreated and treated cells. Data are shown as mean ± SEM of three independent experiments, each in triplicate (n = 3). (TIF) [file ppat.1006247.s001.tif]

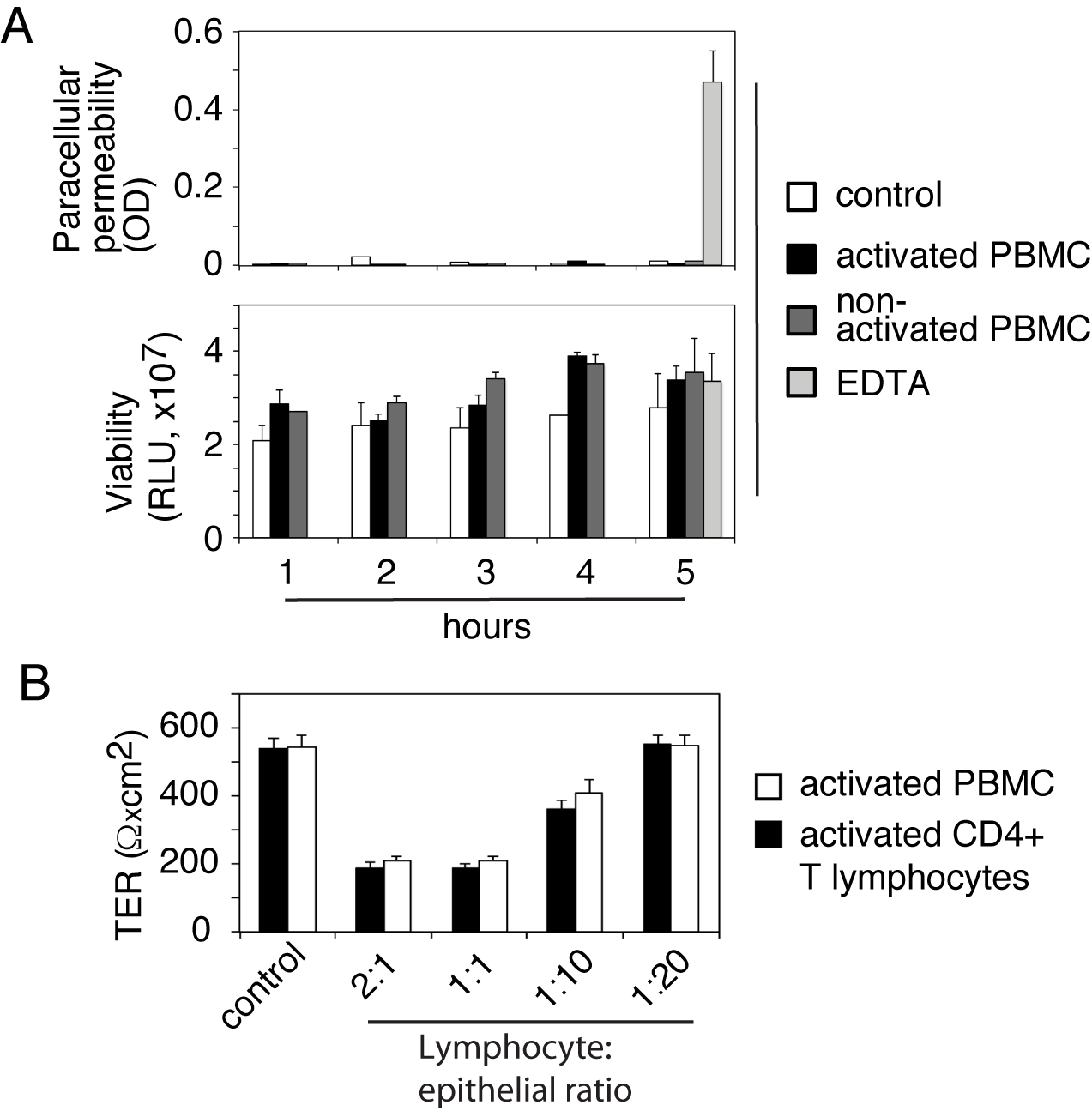

Supplement: S2 Fig — (A) Activated and nonactivated PBMC were added to the AP surface of polarized tonsil epithelial cells, and after 1, 2, 3, 4 and 5 h the paracellular permeability (upper panel) and cell viability (lower panel) were examined. As a control, one set of polarized tonsil cells were treated with 10 mM EDTA for 30 min. (B) Activated PBMC and CD4+ T lymphocytes were added to the AP surface of polarized tonsil epithelial cells at lymphocyte–epithelial ratios of 1:1, 1:2, 1:10 and 1:20. After 4 h TER was measured. (A, B) Data are shown as mean ± SEM of three independent experiments, each in triplicate (n = 3). (TIF) [file ppat.1006247.s002.tif]

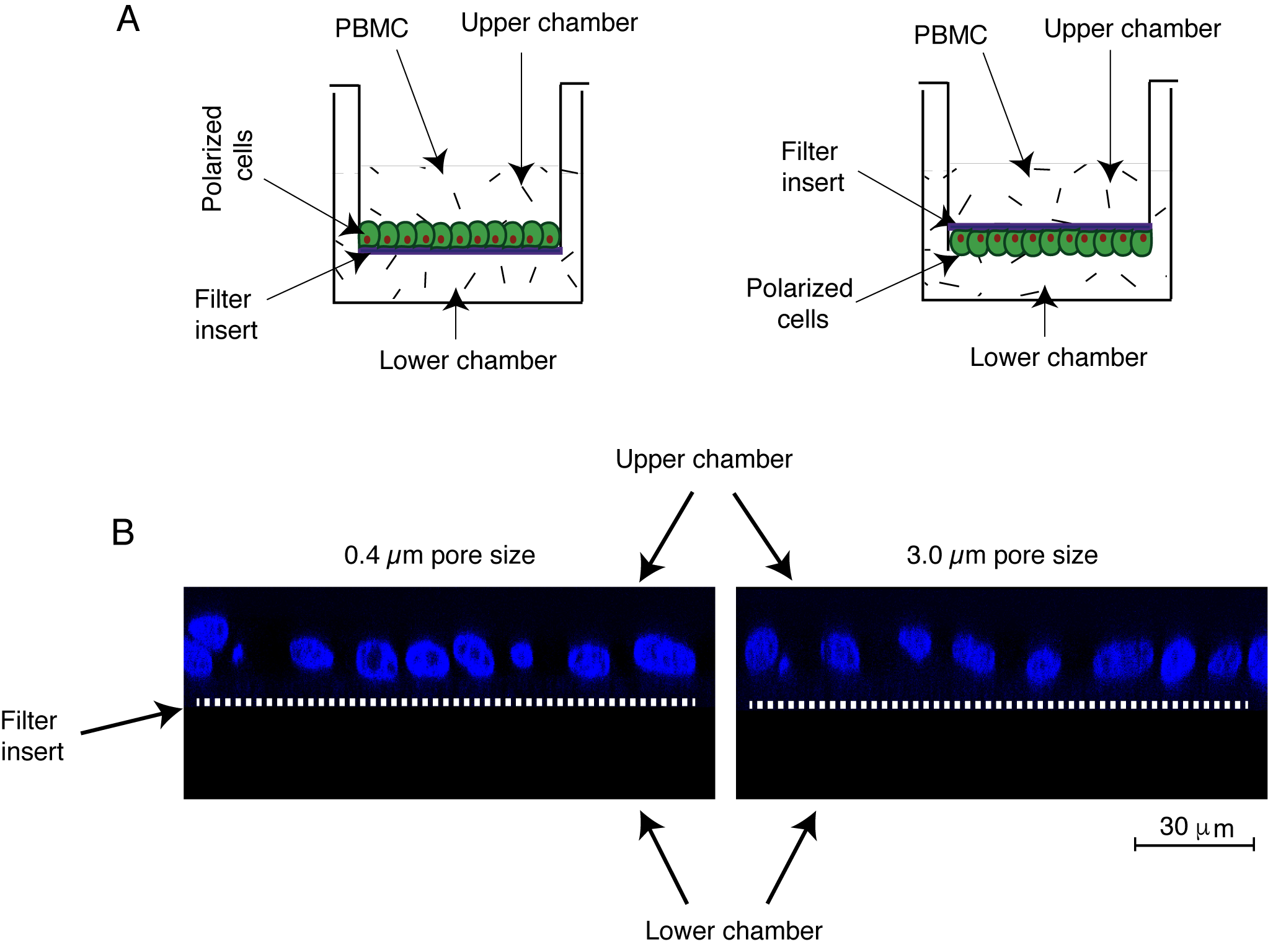

Supplement: S3 Fig — (A) To cocultivate PBMC with AP membranes of polarized epithelial cells, cells were grown on the upper surfaces of Transwell filter inserts, with AP membranes facing upward. To cocultivate PBMC with BL membranes of polarized epithelial cells, cells were grown on the lower surfaces of Transwell filter inserts, with AP membranes facing downward. Addition of PBMC to the upper chambers of the Transwell inserts allowed binding of lymphocytes to the AP or BL surfaces of polarized cells. (B) Tonsil epithelial cells were seeded into the upper chamber of Transwell inserts with 0.4-μm (left panel) and 3-μm (right panel) pore sizes. After 12 days cells were fixed and cell nuclei were stained with TO-PRO-3 iodide (blue). Cells were analyzed by confocal microscopy by x-z vertical planes. Similar data were obtained in three independent experiments using tonsil, foreskin and cervical epithelial cells. (TIF) [file ppat.1006247.s003.tif]

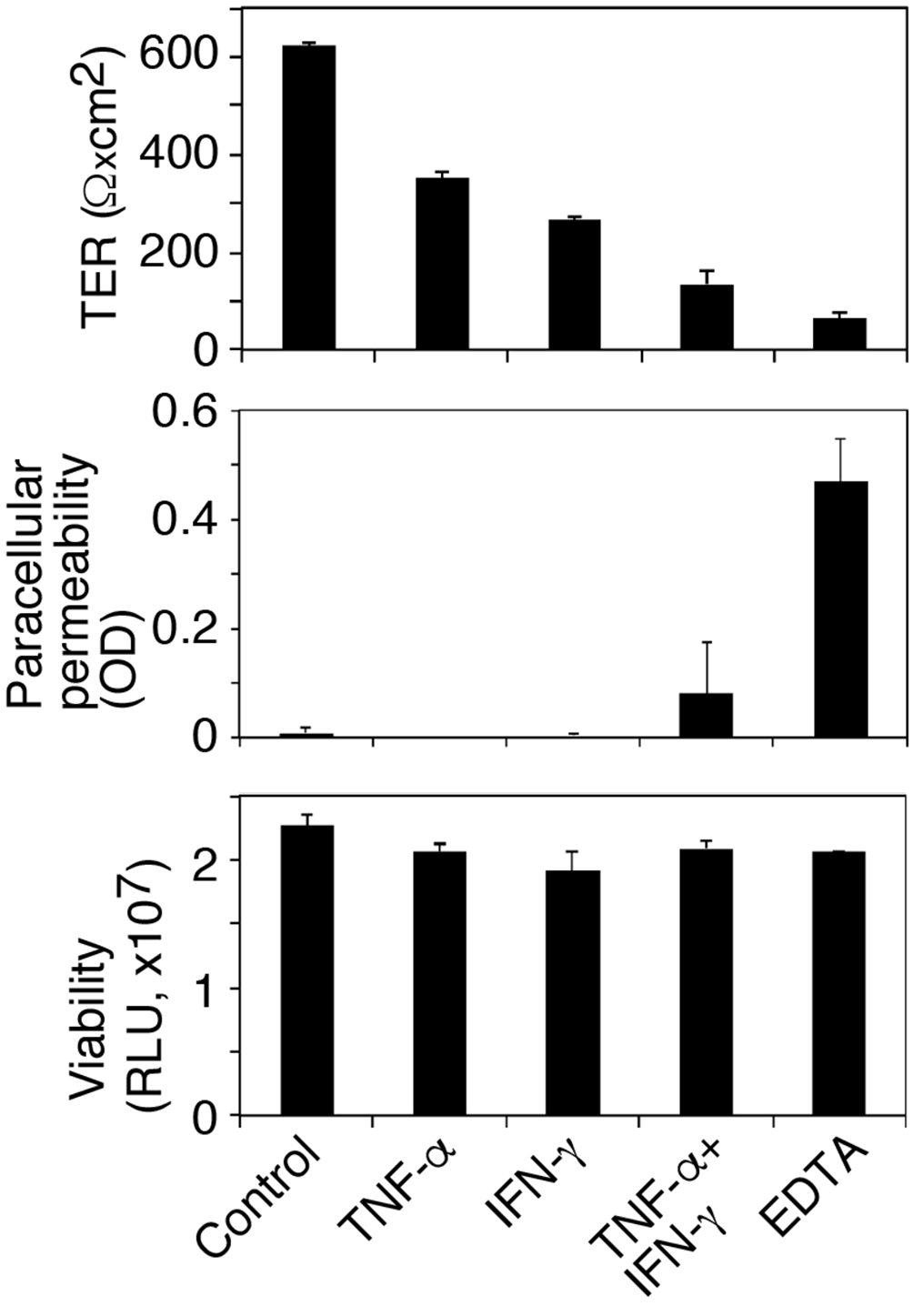

Supplement: S4 Fig — Polarized tonsil epithelial cells were treated with recombinant TNF-α and IFN-γ alone and in combination for 24 h. Then, untreated (control) and cytokine-treated cells were examined for TER (upper panel), paracellular permeability (middle panel) and cell viability (lower panel). Data are shown as mean ± SEM of three independent experiments, each in triplicate (n = 3). (TIF) [file ppat.1006247.s004.tif]

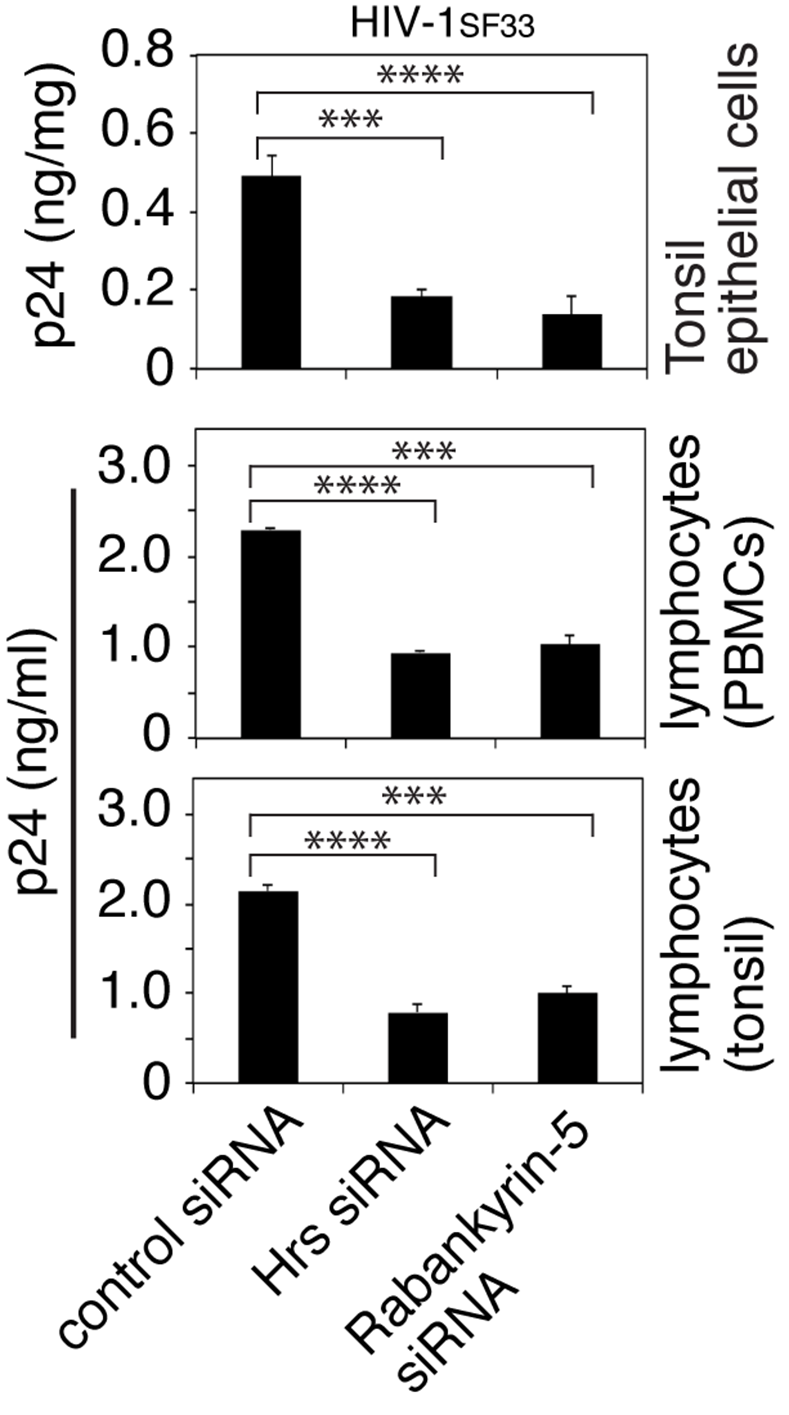

Supplement: S5 Fig — Polarized tonsil cells were transfected with control siRNAs or siRNAs against Hrs and rabankyrin-5 and after 72 h were exposed to HIV-1SF33. After 3 days, one set of siRNA-transfected cells was examined for intracellular virus (upper panel). The next sets of siRNA-transfected cells were cocultured with activated CD4+ T lymphocytes isolated from PBMC (middle panel) or tonsil tissues (lower panel). Four hours later, lymphocytes were collected and grown for 4 days, and virus infection was examined by ELISA p24. Data are shown as mean ± SEM of three independent experiments, each in triplicate for each experimental condition (n = 3). ***P < 0.0001 and ****P < 0.00001, compared with the control siRNAs. (TIF) [file ppat.1006247.s005.tif]
